# The influence of Cenozoic Eurasia-Arabia convergence on the Southeast Arabian Foreland Basin: new geochronological and geochemical constraints from syn-kinematic carbonate mineralization

**DOI:** 10.1038/s41598-023-31611-x

**Published:** 2023-03-16

**Authors:** Francesco Arboit, Kerstin Drost, Alessandro Decarlis, David Chew, Dominik Hennhoefer, Andrea Ceriani

**Affiliations:** 1 Department of Earth Sciences, Khalifa University of Science and Technology, Abu Dhabi, United Arab Emirates; 2 Research and Innovation Center on CO2 and H2 (RICH), Khalifa University of Science and Technology, Abu Dhabi, United Arab Emirates; 3 Department of Geology, School of Natural Sciences, Trinity College Dublin, Dublin 2, Ireland

**Keywords:** Tectonics, Geology, Structural geology, Geochemistry

## Abstract

The Cenozoic succession of the Jabal Hafeet anticline yields the most complete surface expression of the deformation that affected the Southeast Arabian Foreland Basin (SEAFB). The carbonate rocks of the Eocene Rus Formation comprise the core of the Jabal Hafeet anticline and host a network of fractures and carbonate veins associated with dynamic fracture opening and sealing events. These fracture networks developed during the propagation of compressional stresses from the Makran and Zagros fold-and-thrust belts into their foreland basin system (the SEAFB) and are associated with Arabia-Eurasia convergence. Syn-kinematic calcite veins associated with the Cenozoic folding events in the SEAFB were dated by U–Pb LA-ICP-MS carbonate geochronology and characterized further by Raman fluid-inclusion geochemistry. The U–Pb data show that Cenozoic compression linked to the propagation of the Makran fold-and-thrust belt into the SEAFB took place from c. 20 Ma (early Miocene) to c. 2 Ma (mid Pleistocene). Raman fluid-inclusion data reveal the presence of complex hydrocarbons within the parent carbonate-bearing fluids, reflecting a fluid transport pathway between the upper Cenozoic rocks and deeper hydrocarbon-bearing Mesozoic sequences. Combined isotopic and geochemical datasets show that the deformational history of the SEAFB is likely related to the reactivation of inherited deep-seated structures in the upper Cenozoic stratigraphic sequence due to the far-field stress propagation from the Makran belt into the Arabian peninsula, rather than the propagation of a thin-skinned deformation architecture.

## Introduction

Paleostress reconstruction analysis is of increasing importance as it can be applied to a diverse suite of fields, including the sustainable exploration and exploitation of resources and studies on the potential of reservoirs and storage sites (e.g. CO_2_ storage and nuclear waste repositories). A good understanding of the subsurface geology, the stress history and associated fracture and fault networks is key for paleostress analysis. Additionally, understanding the timing and nature of the deformation history within foreland basins is of crucial importance for geodynamic reconstructions of convergent orogens^1^. Syn- and post-tectonic carbonate veins that form in foreland belts represent a robust proxy to obtain such data, with the U–Pb age of the carbonate cements and the geochemical composition of the fluid inclusions yielding information on the timing of the deformation-mineralization event as well as the composition of the parent fluids.

**Figure 1:**
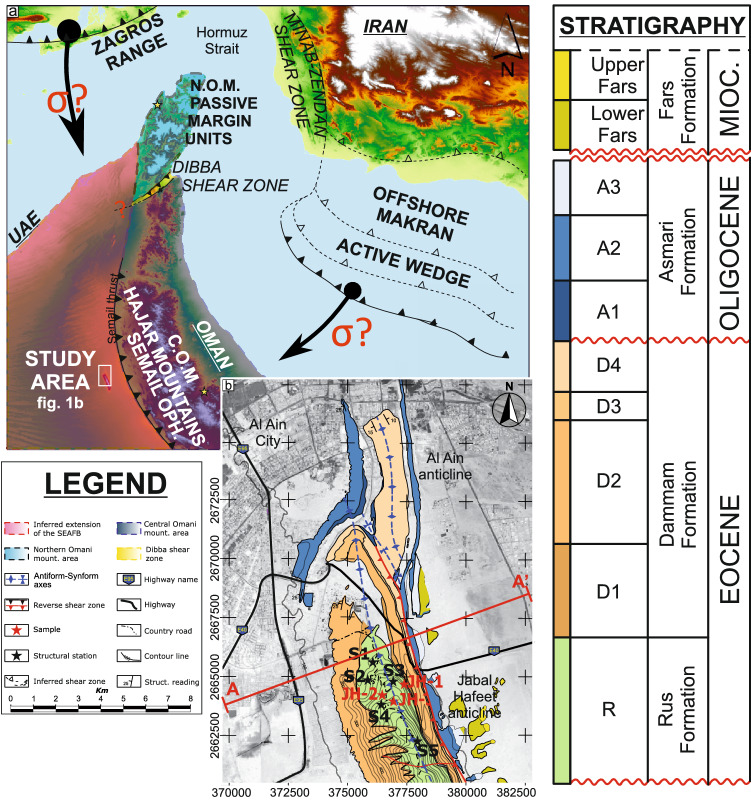
(**a**) Digital Elevation Model (based on SRTM satellite images, created in ArcMAP 10.6.1, ESRI Spatial Analyst) of the area between Iran, Oman, United Arab Emirates with a schematic representation of the major tectonic lineaments (adapted from^2,3^) and geological domains (adapted from^2,16^) in the region. Yellow stars indicate the location of the carbonate U–Pb ages from^6,7^. (**b**) Schematic geological map of the Cenozoic units outcropping in the Jabal Hafeet anticline near the city of Al Ain, showing the location of the structural measurement stations and the sample collection sites. Base-map consists of a Landsat 8 satellite photo of the area using the WGS84 geodetic datum.

The convergence of the Arabian and Eurasian plates led to the closure of the Neotethys Ocean in the late Eocene^2^, and resulted in the propagation of stresses from the Zagros and Makran fold-and-thrust belts into their foreland basin system—the southeast Arabian foreland basin (or SEAFB, Fig. 1). These two fold-and-thrust belts developed adjacent to each other on the Eurasian Continent and are separated by the Zendan transcurrent fault zone, east of the Arabian Peninsula (Fig. 1a).

The convergence of the Arabian and Eurasian plates led to the development of two main tectonic domains on the SE Arabian peninsula: the Permo-Mesozoic Arabian passive margin domain in the northern Oman mountains and a structurally higher domain represented by the continental slope/basinal sediments and Semail Ophiolites of the Central Oman mountains to the south (Fig. 1a). These two tectonic domains are separated by the NE-SW striking Dibba shear zone, which lies parallel to the Hormuz Strait Syntaxis^4^, and appears to have developed as a continuation of a continent-ocean transform fault zone. The Dibba shear zone has linked to a number of faults across the Gulf of Oman that separate the Zagros from the Makran collisional belts (e.g. the Zandan shear zone in Fig. 1a) ^4^. Previous authors^4,5^ have postulated that the Dibba shear zone accommodated tectonic progression of the syntaxis between the Zagros and Makran fold-and-thrust belts; however, direct evidence has yet to be found.

Recent U–Pb dating of syn-kinematic calcite veins^6^ has shown that late Mesozoic carbonates of the northern Oman mountains to the NW of the Dibba shear zone have undergone a polyphase tectonic evolution, which involved top-to-the-west thrusting at c. 70 and 60 Ma and reactivation of thrusts in the Miocene (c. 13 Ma). In contrast, U–Pb ages from carbonate veins of the central Oman mountains to the SE of the Dibba shear zone indicate shortening-related deformation at c. 64, 40, 33, 22, 16, 7 and 2 Ma^7^. This apparent diachronism between the deformation history of the northern and central Oman mountains is likely due to the different stress regimes of the Zagros and southern Makran fronts propagating into the Arabian peninsula and consequently into the SEAFB, and raises questions on the timing and origin of the driving forces that led to the deformation of the SEAFB^8,9^.

Our combined LA-ICP-MS and Raman datasets from carbonate veins of the Jabal Hafeet anticline (adjacent to the city of Al Ain in the Emirate of Abu Dhabi, Fig. 1b) provide constraints on the origin of the fluids that led to carbonate mineralization in the early Eocene (c. 56 to 48 Ma^8^) Rus Formation, and on the timing of deformation within the SEAFB in the Cenozoic. Beyond that, this case study on the Cenozoic carbonate veins from the SE Arabian Peninsula highlights the potential of this conceptual and methodological approach for unravelling multi-phase tectonic histories of orogenic (carbonate-dominated) forelands.

## SEAFB geological framework

Several tectonic mechanisms have been put forward to explain the driving forces that lead to the formation of foreland basins, either by the surface load in front of accretionary prisms (i.e. topographic), or by subsurface (i.e. buried) loading (e.g. ophiolite obduction^10^). Previous studies^11–14^ have suggested that the SEAFB developed due to the flexural loading of the underlying rifted continental margin by the obduction of Neo-Tethyan oceanic crust in the late Cretaceous^15^. The SEAFB stratigraphic sequence is c. 4 km thick^13,16^ and formed at the leading edge of the obducted allochthonous units over the Arabian passive margin (Fig. 1a). The SEAFB stratigraphic sequence commenced with the deposition of late-Cretaceous carbonate mudstones to rudstones of the Fiqa, Juwaiza and Simsima formations following Semail Ophiolite emplacement at c. 95.5 ± 0.5 Ma^17^, and ended with the deposition of the Cenozoic Rus, Dammam, Asmari and Fars formations^12,18,19^.

The Jabal Hafeet anticline (Fig. 1b) developed within the easternmost SEAFB and is an east-verging double-plunging periclinal fold, with a fold axis dipping c. 40°/250°, trending NNW–SSE for over c. 20 km. It is arranged in a right-stepping en-échelon array together with the minor Al-Ain anticline (Fig. 1b)^8^. The compressive stress field led to folding and uplift of the Jabal Hafeet structure and resulted in exhumation of a complex sequence of carbonate units from the oldest Eocene Rus Formation in the core to the youngest Miocene Fars Formation on the faulted eastern limb^20^. The exposed Cenozoic sedimentary sequence that makes up the Jabal Hafeet structure lies on top of the early Paleogene Umm Er Radhuma and Muthaymima formations, which developed above the Maastrichtian Aruma erosional surface^21^. This late Cretaceous erosional surface developed due to westward progression (present-day orientation) of the flexural forebulge during the initial stages of the obduction of the Semail Ophiolite^11^, and covers the underlying Permian to Cretaceous continental shelf carbonates (Hajar Supergroup).

The timing of deformation in the Jabal Hafeet structure has been disputed, with early stages of compression viewed as synchronous with sedimentation of the Rus Formation in the mid Eocene^22^, or early deformation interpreted as a result of post-Miocene compression^13,20^.

On a regional scale, the structural history of the early Eocene to late Miocene rocks within the SEAFB has been divided into up to four main paleostress stages^23,24^,. These stress histories are characterized by the set in of early compressive stress regimes with SH_MAX_ gradually migrating from an early E–W to a late N–S orientation followed by a final NE-SW oriented extensional stage^23,24^. These tectonic events were associated with the development of shear zones whose orientations are consistent with the systematic N75W and N45E conjugate fault zones that control fluid flow within the SEAFB basement^25^. These inherited shear zones are likely linked to the Ediacaran Najd Fault System that developed as a set of continental transform faults in response to a major episode of late Precambrian extension and continental crust formation in northernmost Afro-Arabia^26^.

The genesis and timing of the carbonate mineralization seen throughout the ophiolite of the central Oman Mountains has been intensively studied in the past decades^27^. However, the origin of the fluids that led to the crystallization of the carbonate veins within the SEAFB has not attracted the same amount of attention. Recent Sr isotope studies on the Cenozoic syn-kinematic carbonate mineralization within the SEAFB has yielded ^87^Sr/^86^Sr values of c. 0.7076–0.7083^24^, which are slightly more radiogenic than the ^87^Sr/^86^Sr signature of Cenozoic and Cretaceous seawater (c. 0.7072–0.7074^28^). The same carbonate veins yield oxygen and carbon isotope values consistent with a burial-uplift geodynamic history^24^, which implies the possible involvement of continental fluids affecting the SEAFB during burial in the Eocene. However, the origin of the fluids, as well as the timing and geodynamic evolution of the SEAFB are still a matter of debate.

## Materials

**Figure 2:**
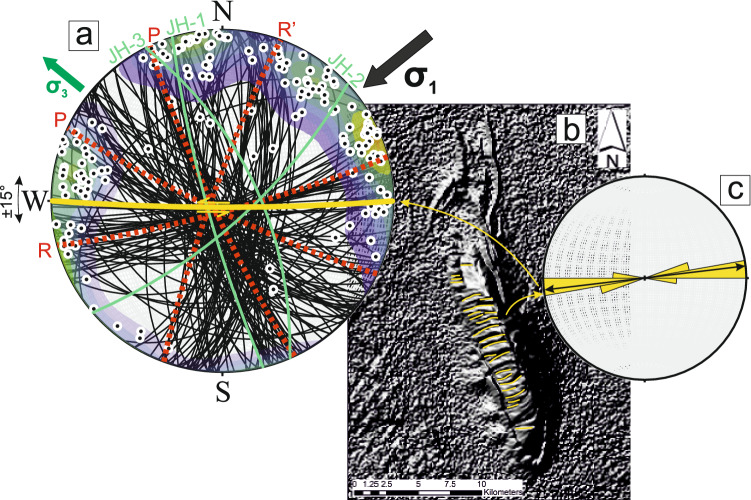
(**a**) Lower-hemisphere equal-area stereonet projection of shear planes with more than 1 mm of carbonate cement infill (N = 166, black lines). Red dashed great circles represent a schematic Riedel-type model constructed using the major structural trends identified by stereographic projection of these shear planes. Black and green arrows indicate the possible orientation of the paleo-maximum (σ_1_) and minimum (σ_3_) horizontal stresses. The yellow great circle indicates the likely orientation of the major strike-slip shear planes based on the orientation of the major drainage lineaments of the Jabal Hafeer anticline, green great cricles represent the orientation of the veins where samples have been collected. (**b**) Hillshade imaging of Jabal Hafeet based on the SRTM digital elevation model of the area showing the orientation of the drainage pattern (yellow lineaments). (**c**) Rose diagram of the orientation of the drainage patterns showing the likely orientation of major strike-slip structures within the Jabal Hafeet anticline.

Structural analysis in this study of the Jabal Hafeet anticline employs c. 500 measurements made on fractures and shear planes (Fig. 2) throughout the Eocene Rus Formation (Fig. 1b). These measurements were undertaken to constrain the general orientation of the principal paleostress axes, and to thus resolve the possible paleostress history that affected the SEAFB Cenozoic units. Three representative calcite and dolomite cements infilling compressive (sample JH-1), transtensive (sample JH-2) and extensive (sample JH-3) shear planes respectively were collected for fluid-inclusion Raman studies and U–Pb dating (Fig. 1b; coordinates in supplementary material).

**Figure 3:**
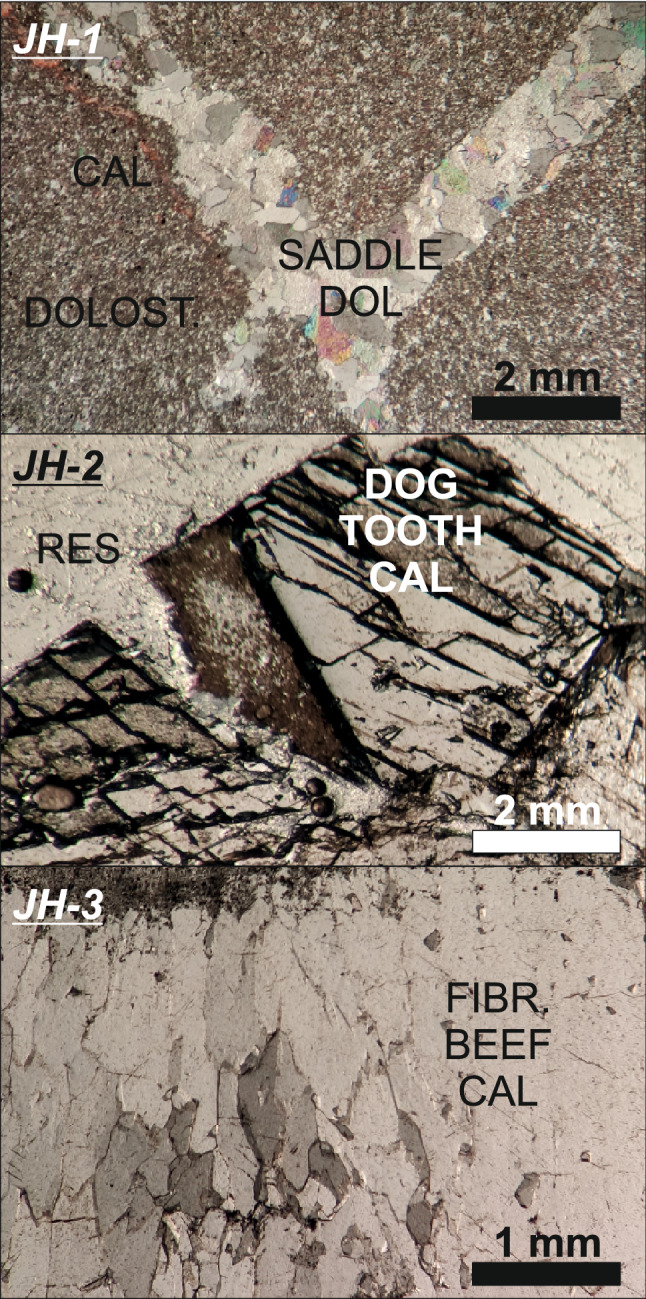
Transmitted (plane-polarized light) thin-section photomicrographs of samples JH-1, JH2 and JH-3. *CAL* calcite, *DOLOST* host rock dolostone, *DOL* dolomite, *RES* resin, *FIBR. BEEF CAL* fibrous beef calcite.

Sample JH-1 was collected from a centimeter-thick carbonate vein within a shear plane with a 85°/252° orientation (Dip/Dip Direction) filled with saddle dolomite cement within the host dolostone (Fig. 3), which was observed in the field to represent one of the earliest structural events. The compressive shear plane infilled by the saddle dolomite cements in sample JH-1 is post-dated by several vein infill events, which include blocky calcite, fibrous calcite, dog tooth calcite and drusy calcite cements. Sample JH-2 was collected from a transtensive open vein oriented 78°/138°, and is characterized by the presence of prismatic blocky calcite/centimetric dog-tooth calcite cements. Sample JH-3 was collected from one of the latest extensional shear events that developed during the uplift of the structure^8^. It is oriented 62°/177° and is comprised of a millimetric layer of fibrous beef calcite (Fig. 3).

## Results

### Fracture data processing

Regional fracture sets were identified based on shared orientation trends from c. 500 fractures measured at five separate localities within the Rus Formation (Fig. 1b, coordinates in supplementary material), and on c. 100 faults with relative kinematic indicators measured throughout the Rus Formation (shear projection in supplementary material). A large number of veins thicker than 1 mm (Fig. 2) were identified as shear planes^29,30^, and the projection of these shear planes appeared to converge on the same regional trend after isolating data by orientation and removing the dip of bedding by stereographic rotation around the Jabal Hafeet anticlinal axis. The compressive deformational event is composed of four sets of transpressional and compressive shear features oriented c. 80°/160°, 85°/280°, 85°/250°, 80°/220°. These earliest sets of shear planes were followed by a younger pervasive swarm of c. E-W striking conjugate extensional shears^8^ (faults projection in supplementary material).

### Raman analysis

**Figure 4:**
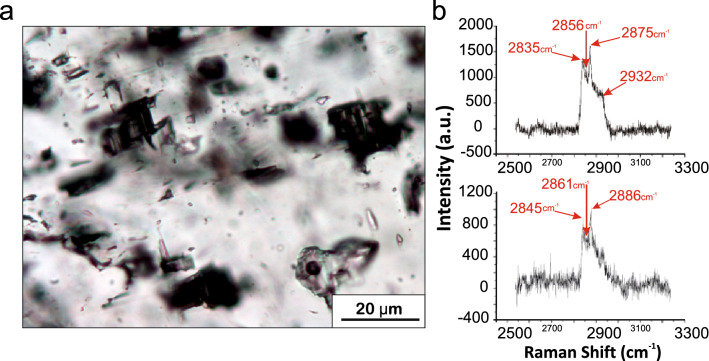
(**a**) Image of one (gas) and two (gas + liquid) phases fluid inclusions in sample JH-2. (**b**) Raman spectra showing evidence of the presence of hydrocarbons within samples JH-1 and JH-2.

Raman analysis were performed on fluid inclusions (Fig. 4a) within the early compressive equant dolomite crystals (sample JH-1) and the late transtensive clear dog-tooth calcite crystals (sample JH-2). The acquisition of the inelastic scattering from the fluid inclusions revealed a strong signal in the Raman band region of 2800–2950 cm^−1^, which are correlated with C–H stretching modes within Raman-active fluids at those frequencies (Fig. 4b).

### LA-ICP-MS U–Pb dating and trace element mapping

**Figure 5:**
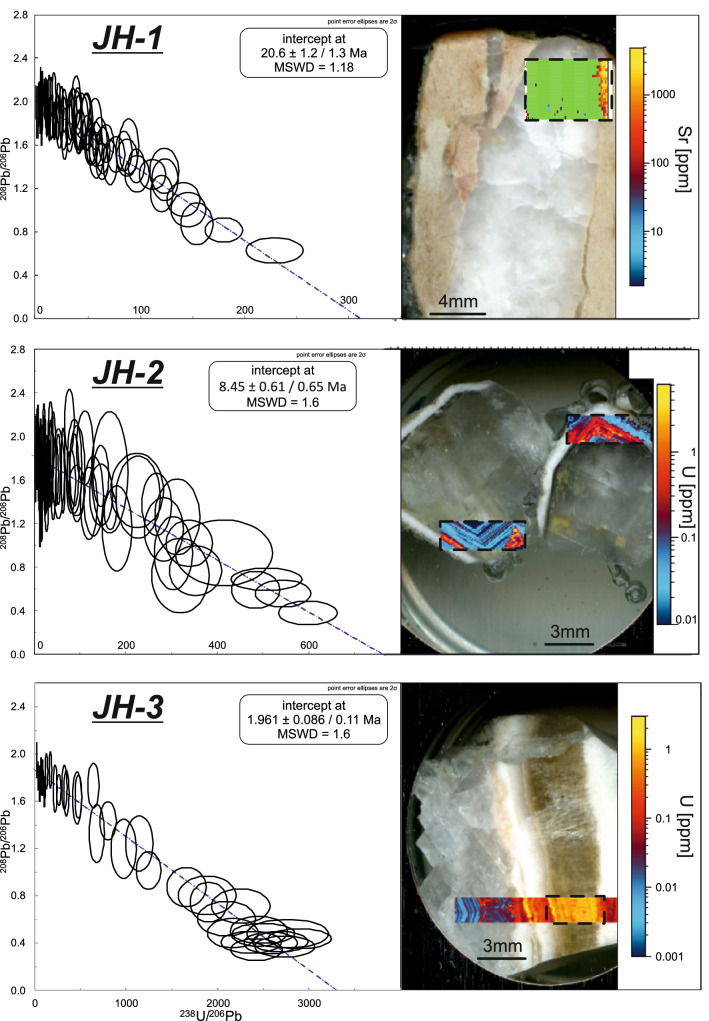
^208^Pb/^206^Pb vs ^238^U/^206^Pb age plots (86-TW space) of samples JH-1, JH-2 and JH-3. The elemental concentration maps (strontium and uranium ppm) are superimposed on the vein cement images and denote the areas dated by LA-ICP-MS.

Three carbonate vein samples from the Rus Formation in the core of the Jabal Hafeet anticline yield sufficient U and radiogenic Pb for determining U–Pb dates. Sample JH-1 is a dolomite vein collected from the damage zone within the footwall of one of the earliest steep-dipping transpressive shear planes. Sample JH-1 yields a U–Pb date of 21.4 ± 2.3/2.4 Ma (MSWD = 1.20), while a repeat analysis on a larger area of the same vein yields a date of 20.6 ± 1.2/1.3 Ma (MSWD = 1.18) (Fig. 5). Sample JH-2 is a vein filled with ‘dog-tooth’ calcite crystals collected from the damage zone of a steeply-dipping strike-slip shear plane reactivated as an open fracture joint. The calcite cement yields a date of 8.88 ± 0.44/0.51 Ma (MSWD = 1.19) with a repeat analysis yielding a date of 8.45 ± 0.61/0.65 Ma (MSWD = 1.6) (Fig. 5). Sample JH-3 was collected from a vein in the damage zone of a normal shear plane that has recorded multiple shear/opening events. The second episode of shear reactivation recorded within the normal fault comprises a layer of brown fibrous beef calcite crystals. The analysis of this layer of calcite yields a U–Pb date of 1.912 ± 0.075/0.095 Ma (MSWD = 1.21) with a repeat analysis of 1.961 ± 0.086/0.11 Ma (MSWD = 1.6) (Fig. 5).

**Figure 6:**
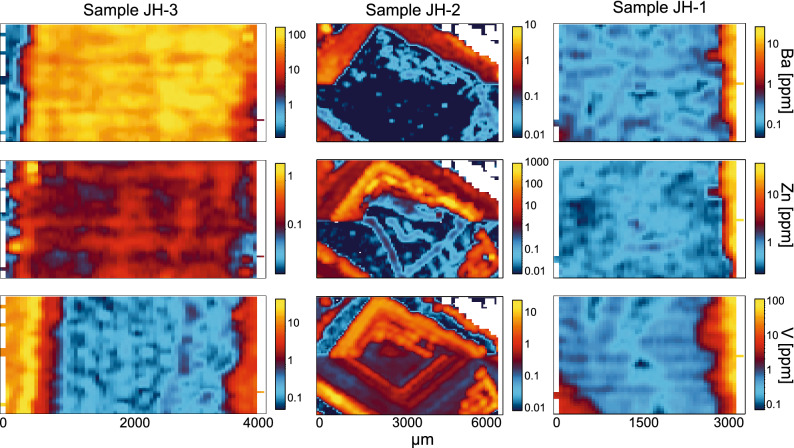
Images showing the elemental concentration (ppm) of barium, zinc and vanadium in samples JH-1, JH-2 and JH-3. Location of the elemental concentration maps are denoted by the black dashed boxes in Fig. 5.

The LA-ICP-MS U–Pb dating approach in this study employs a mapping technique that enables simultaneous acquisition of major and trace element data. Key major and trace elements that are sensitive to the original fluid composition, detrital components (e.g. Rb, Ga, V, Zn), post-formational fluid ingress, mineralogical changes, or diagenetic overprinting (Drost et al., 2018) were also acquired. Dolomite cement JH-1 yield a distinctive low concentration of barium (< c. 0.5 ppm), zinc (< c. 1 ppm) and vanadium (< c. 3 ppm) (Fig. 6). In contrast, the calcite vein cements in samples JH-2 and JH-3 are characterized by large sections of the crystals yielding highly variable concentrations of these metals with barium up to > 100 ppm in JH-3, and zinc up to 1000 ppm and vanadium up to 10 ppm in JH-2 (Fig. 6; supplementary material).

## Discussions

### Origin of the mineralizing fluids

Recent numerical modelling studies indicate stylolites are one of the main fluid sources and fluid migration pathways in carbonates^31^. The Eocene Rus Formation presents little to no evidence of either burial or tectonic stylolites^8^, which raises the question on the origin of the carbonate fluids driving the overpressure within the shear zones in the Rus Formation. Previous petrographic and isotopic investigations on the carbonate syn-kinematic cements throughout the Jabal Hafeet anticline^24^ have revealed a possible paragenetic history of carbonate mineralization that started with the crystallization of dolomitic cements in a phase of burial diagenetic alteration, which was then followed by late crystallization of fibrous and dog-tooth calcites^24^. This diagenetic sequence implies a burial origin for the early Miocene dolomitic cements (saddle dolomite crystals such as in sample JH-1) (δ^18^O_VPDB_ −12‰, δ^13^C_VPDB_ −1‰ ), and a meteoric origin for the late Miocene and Pleistocene cements (prismatic dog-tooth and fibrous calcite crystals such as in samples JH-2 and JH-3) (δ^13^C_VPDB_ −12‰).

The ^87^Sr/^86^Sr compositions of the carbonate rocks and cements throughout the Cenozoic units of the Jabal Hafeet anticline exhibit values from 0.70766 to 0.70832^24^, with the earliest dolomite veins yielding the most positive δ^13^C values and the least radiogenic Sr values of c. 0.70775. The mean ^87^Sr/^86^Sr composition of the Cenozoic carbonate cements from the veins cutting the Jabal Hafeet structure is slightly more radiogenic (c. 0.708) than Cretaceous and Cenozoic seawater (i.e. c. 0.7072–0.7074^28^), and is much more radiogenic than fluids derived from a mafic source such as the Semail ophiolite (c. 0.703^32^). However, the origin of the fluids that led to early Miocene carbonate mineralization within the Rus Formation is still a matter of debate.

Mechanical (e.g. molecular shape, flow rate, and metal concentration) and physiochemical (e.g. ionic strength, Eh and pH) parameters are the main factors controlling the solubility and transport of heavy-metal ions^33^. The dog-tooth calcite cement in sample JH-2 is characterized by concentrations of redox-sensitive elements such as V and Zn of up to c. 10 and 1000 ppm respectively, whereas the dolomite and fibrous beef calcite cements in samples JH-1 and JH-3 yield concentrations less than c. 1 ppm for both heavy metals (Fig. 6). These concentration differences are evidence either of different Eh–pH conditions at the time of crystallization, or different chemical compositions in the parent fluids that led to the crystallization of the different generations of carbonate veins. However, sample JH-3 is also characterized by Ba concentrations > c. 100 ppm, whereas Ba is < c. 0.1 ppm in the JH-1 dolomite sample. Previous studies have observed that the solubility of Ba contrasts with that of transition metals such as V and Zn, and increases in reduced environments^34^. These data do not resolve the redox conditions during the crystallization of the JH-1 dolomite, but imply that low Eh conditions were likely during the crystallization of the JH-3 fibrous beef calcite. As silicate and sulphate weathering are the likely primary control/source on the concentrations of heavy metal ions in fluid solutions^35^, it is likely that the JH-1 dolomite crystallized from fluids that neither originated nor underwent ion exchange with silicate or sulphate-bearing rocks.

In addition to major calcite and dolomite peaks around the c. 1080 and 1100 cm^−1^ Raman bands, the Raman spectrum of fluid inclusions from the c. 20 and 8 Ma cement phases in the Rus Formation is characterized by minor peaks in a narrow spectral interval between the 2800–2950 cm^−1^ Raman bands. This indicates the presence of methane and complex hydrocarbons in the original fluid that led to the crystallization of at least the early and late Miocene tectonic cements in the Rus Formation^36^. Previous studies^37^ has shown that Raman spectral bands are affected by hydrocarbon molecular structure and base groups rather than by carbon concentration, and the Raman spectra of fluid inclusions within the early carbonate vein systems are similar to the signal of C_n_H_2n+2_ saturated hydrocarbons. Thus, the Raman spectra of the inclusions confirm the presence of complex hydrocarbons in addition to methane during the early Miocene fluid flow episode.

**Figure 7:**
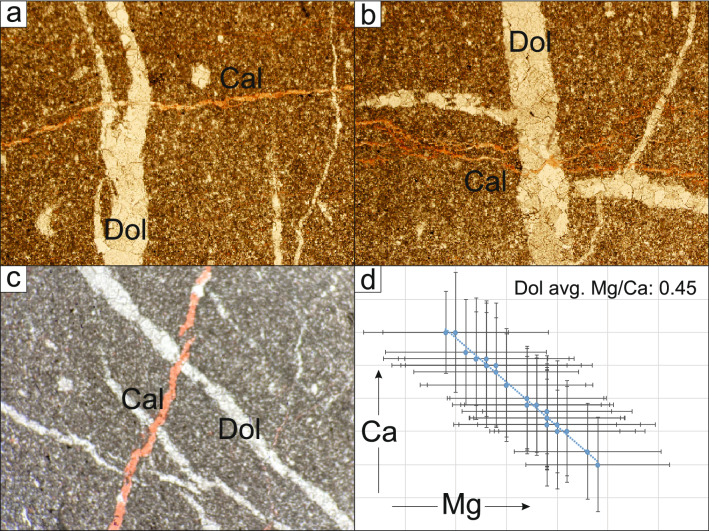
(**a–c**) Transmitted (plane-polarized) light thin-section images of sample JH-1 stained with red-alizarin. (**d**) Calcium vs magnesium ratio of dolomite sample JH-1.

**Figure 8:**
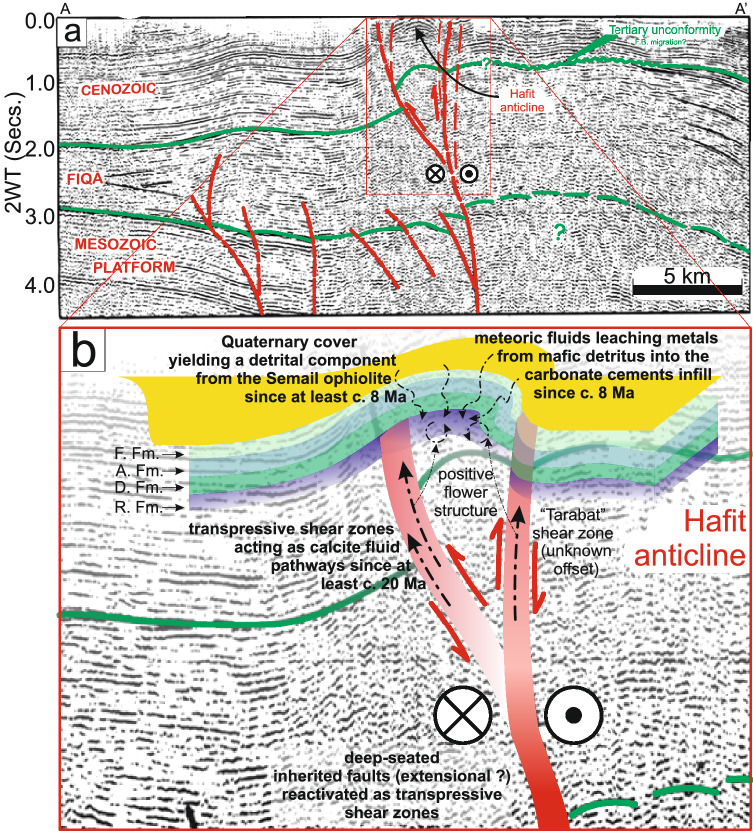
(**a**) SW-NE orientated seismic transect across the Jabal Hafeet structure (modified from^13^, position of the seismic line given on Fig. 1b). Solid and dashed red lines showing the position of transpressive shear zones (interpretation from^13^). Green lines denote stratigraphic horizons between the underlying Mesozoic platform (Upper Cretaceous Fiqa Formation) and the overlying Cenozoic units (interpretation from^13^). (**b**) schematic model of the Cenozoic deformation within the Jabal Hafeet anticline caused by shallow folding linking to deep high-angle reverse faults cutting from the Cretaceous into the Cenozoic units. *F. Fm.* Fars Formation, *A. Fm.* Asmari Formation, *D. Fm.* Dammam Formation, *R. Fm*. Rus Formation. Shaded red planes showing the possible location of the transpressive faults that act as pathways for the carbonate-rich fluids (dashed black arrows) that infill shear planes within the Rus Formation. Red arrows show the interpreted kinematics of the transpressive faults.

The absence of diffusive mass transfer (i.e. stylolite seams) within the Eocene Rus Formation, the presence of complex hydrocarbons within the fluid inclusions and the elemental and Sr-isotopic compositions of the earliest carbonate veins (e.g. JH-1) corroborate a parent fluid source from the underlying Cretaceous carbonate units. Petrographic observations and LA-ICP-MS element data from sample JH-1 revealed the presence of near-stoichiometric primary dolomite in the early Miocene vein set (Fig. 7). These dolomite crystals are characterized by a constant Mg/Ca ratio of c. 0.45, confirming earlier models of Mg sourcing from hot basinal fluids (> 4500 m and c. 120°C^24^). Combined, the presence of hydrocarbons and the radiogenic ^87^Sr/^86^Sr composition of the syn-kinematic carbonate cements rule outs a fluid pathway between the SEAFB and the obducted ophiolite in the central Oman mountains via a low angle detachment^2^ (Fig. 8).

The negative δ^13^C values of the cements infilling the later shear planes^24^, the low V, Zn and high Ba concentrations in sample JH-3, and the high concentrations of heavy metals in sample JH-2 support a scenario, since at least the late Miocene, of variable redox conditions and the influence of meteoric fluids that were transporting metallic ions. It is possible that the source fluids underwent ion exchange with weathered silicate and sulphate material from the neighboring obducted ophiolites, which were uplifted at c. 30 Ma^38^ and were likely already being eroded above the SEAFB^39^ prior to the crystallization of JH-2 vein calcite at c. 8 Ma (Fig. 8).

### The SEAFB and Jabal Hafeet structural evolution

#### Early Miocene

The Cenozoic carbonate sequence of the Jabal Hafeet structure is effectively unmetamorphosed and was deformed within 5 km of the surface^8^, and thus a principal stress must be vertical^40^. Our analysis of the shear plabes that deformed the Rus Formation satisfies these criteria. They also agree with recent structural interpretations^8^, which attributed the structural features within the Rus Formation to syn-folding deformation under a protracted c. ENE-WSW compressive stress field (Fig. 2), as opposed to previous interpretations that favored a sequence of different paleo-stresses deforming the Eocene units^23,24,41^. Our interpretation is also consistent with continuous horizontal shortening in the SEAFB arising from propagation of ENE-WSW compression from the neighboring central Oman mountains (Fig. 1a). Recent models for the tectonic evolution of the central Oman mountains have employed low-temperature thermochronology to constrain the timing of uplift. Four main uplift phases have been detected from c. 70 to 20 Ma^38^, with the main folding and doming/uplift stage in the central Oman mountains constrained to between c. 40 and 20 Ma.

However, the lack of any absolute time constraints on the SEAFB deformation history has not allowed a general consensus to be reached on the timing and origin of deformation in the SE Arabian foreland. Early models proposed that folding in the Jabal Hafeet anticline initiated in the mid Eocene and had terminated by the early Miocene^22,23,42,43^, while more recent tectonic reconstructions of the Jabal-Hafeet structure have proposed a mid to late Miocene timing of deformation in the SEAFB^8^. However, the earliest evidence of deformation within the Cenozoic stratigraphic sequence consists of an angular unconformity (a minor discordance in bedding dip of c. 10°) between the Oligocene Asmari Formation and the unconformably overlying Miocene Fars Formation, which implies that folding of the SEAFB was active at least by c. 20 Ma^44^. This early Miocene angular unconformity between the underlying Asmari and overlying Fars formations overlaps in age with the oldest U–Pb date collected from sample JH-1 in a syn-kinematic compressive shear event observed within the Rus Formation at 20.6 ± 1.2/1.3 Ma (sample JH-1, Fig. 5).

The complex fold evolution of the Zagros belt has been interpreted to have developed in its southern Fars arc during the Miocene^45^, which has been further constrained by U–Pb zircon dating of Arabia-Iran post-collisional volcanism that commenced at c. 15–13.5 Ma^46^. Thus, the lack of Eocene to Miocene stratigraphic constraints in the central Oman mountains^38^ together with the concomitant Miocene onset of collision in the southern Zagros^45,47^ has led to SEAFB deformation being attributed to the propagation of horizontal shortening from the Zagros^2,4,13,18,47,48^.

However, mid Miocene (c. 13 Ma) U–Pb dates recently obtained from syn-kinematic (strike-slip) calcite veins in the northern Oman mountains^6^ and which are likely associated with N–S oriented Zagros compression^6,47–49^, have yet to be documented either in the southern tectonic domains of the central Oman mountains^7^ or the SEAFB. In contrast, deformation in the Makran belt started at c. 23 Ma^50^, which is similar in age to the 22 ± 4 and 21.5 ± 0.5 Ma U–Pb carbonate ages from NE-SW oriented strike-slip shear planes that developed during the main folding phase in the central Oman mountains^7^. These ages are within uncertainty of the 20.6 ± 1.2/1.3 Ma U–Pb date from the JH-1 transpressional calcite vein cement in the Rus Formation, and this coeval deformation allows correlation of shortening within the SEAFB with propagation of c. NE-SW horizontal stress from the Makran belt.

#### Late Miocene

**Figure 9:**
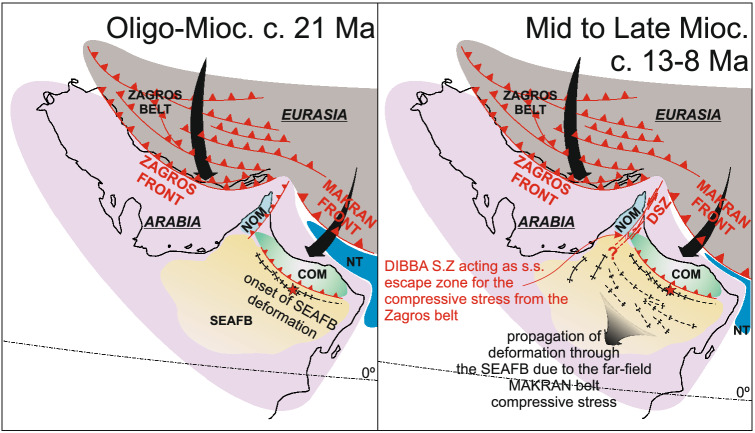
Schematic model of the geodynamic evolution of the Arabia-Eurasia collision from the early to Late Miocene. Position of the continental margins, subduction zones and the tectonic structures in the Zagros and Makran belts and in Iran are from^3,51^. Tectonic structures in the UAE and Oman are from^6,52,53,62^. The extent of the offshore Semail Ophiolite is unconstrained. *NOM* northern Oman mountains, *COM* central Oman mountains, *DSZ* Dibba shear zone, *NT* Neotethys; red star shows the location of the study area.

The two youngest samples date shear structures generated by strike-slip and doming-extensional events in the late Miocene (sample JH-2; 8.88 ± 0.44/0.51 Ma and 8.45 ± 0.61/0.65 Ma) and Pleistocene (sample JH-3; 1.912 ± 0.075/0.095 Ma and 1.961 ± 0.086/0.11 Ma). These shear events indicate the SEAFB went through an episodic compression-induced folding history from c. 20 Ma, which was subsequently dominated by steep transpressive faults reactivated during subsequent phases of gravity driven extensional deformation due to the progression of folding and doming of the Jabal Hafeet structure. This deformation history in the SEAFB is similar to the tectonic evolution of the outer Makran belt and central Oman mountains, with all these tectonic domains having experienced NE-SW oriented compression and uplift at 7–8 Ma and 1.5–2 Ma (^7,52^, this study). Thus our new U–Pb dates support a possible geodynamic scenario whereby the SEAFB accommodated residual stress from N–S directed Eurasia-Arabia convergence along the eastern Makran front through the central Omani mountains (Fig. 9).

Integrating the structural history above with the isotopic and trace element signatures of the veins cements cutting the Rus Formation allows us to envisage a SEAFB structural framework with deep-rooted transpressive shear planes acting as preferential fluid-flow pathways since the early Miocene between the upper Eocene Rus Formation and the oil-bearing Cretaceous units below (Fig. 8). The new U–Pb and geochemical data indicate that the SEAFB deformation was likely induced by far-field transmission of compressive stress from the Makran belt via the neighboring hinterland domains of the central Omani mountains. It is thus possible that the lack of evidence for structures induced by Zagros compression within the Cenozoic units of the SEAFB are due to strike-slip reactivation of the Dibba shear zone, which may have accommodated the N–S oriented Zagros stress-field by acting as a lateral escape zone^6^ (Fig. 9).

## Conclusions

1. The parent fluids of the earliest syn-kinematic dolomite mineralization within the Jabal Hafeet structure are characterized by the presence of complex hydrocarbons that likely originated from deep-seated shear planes that sourced deeper Cretaceous oil-bearing units, and were reactivated during Cenozoic deformation of the SEAFB.
2. Early syn-kinematic dolomite mineralization is dated by U–Pb method at 20.6 ± 1.2/1.3 Ma. This date for the onset of deformation in the SEAFB overlaps in age with the unconformity developed between the Oligocene Amman and the Miocene Fars formations.
3. Subsequent deformation (syn-kinematic calcite mineralization) within the Eocene Rus Formation is dated at c. 8.5 Ma and 1.9 Ma.
4. Our U–Pb carbonate data constrain tectonic activity in the SEAFB from c. 21 to c. 2 Ma and correlate closely with the similar tectonic history of the central Oman mountains, supporting a geodynamic scenario whereby the SEAFB accommodated residual stress arising from of N–S Eurasia-Arabia convergence along the eastern Makran front.
5. Integrating the geochronological, isotopic and geochemical data implies the SEAFB deformation architecture was due to far-field transmission of compressive stress and did not involve propagation of stress along low-angle detachments between the SEAFB and the central Oman mountains.
6. The lack of evidence for structures induced by Zagros compression within the Cenozoic units of the SEAFB may imply that the late Cenozoic N–S oriented compressive Zagros stress field was accommodated by sinistral mid-late Miocene strike-slip reactivation along the Dibba shear zone in the, which acted as a lateral escape zone.
7. This study demonstrates the effectiveness of integrated geochronology and geochemical analysis of syn-kinematic carbonate cements based on elemental and isotopic ratio mapping by LA-ICP-MS with helps determine both the nature of the parent fluids composition and the age of carbonate mineralization associated with specific deformation events.

## Methods

### Raman experiments

Micro‐Raman spectrometry was carried out at the Khalifa University with a WITEC ALPHA 300 RAS system equipped with a He–Ne laser source. Analysis was carried out using 532 nm green light on one-phase two-phase (liquid + vapour) fluid inclusions within sample JH-1 and JH-2. The spectrometer employs two manually switchable lattices (1,800 and 600 R/mm) and a CCD detector (256 × 1024 pixels) with Peltier air circulation cooling. A 100 × air objective was used, and the laser spot was c. 1 μm in diameter. The laser power was carefully controlled to avoid any heating effect on Raman shifts, and a 1800 grooves/mm grating was used, which provided a spectral resolution of c. 0.7 cm^−1^. The Raman shifts were calibrated using a peak centered at 520.7 cm^−1^ from the Si substrate of the standard.

### LA-ICP-MS experiments

Polished rock slabs in 25 mm diameter epoxy mounts were analysed for characteristic major and trace elements and for U and Pb isotopes using an imaging strategy. Analyses were performed at the Department of Geology, Trinity College Dublin, using a Photon Machines Analyte Excite 193 nm ArF excimer laser ablation system coupled to an Agilent 7900 quadrupole ICP-MS.

The general analytical and data processing routine is described in Drost et al. (2018) while specific details on the laser ablation and ICP-MS systems are given in Supplementary Table 1. Data processing was undertaken in Iolite 3.6^54^ including the add-on Monocle (Petrus et al., 2017). NIST614 was used as a primary reference material to normalize both elemental compositions and U–Pb data. The U–Pb data were then matrix-matched using calcite reference material WC-1^55^. One of our samples (JH1) is a sparry dolomite vein, but due to the lack of a suitable dolomite reference material, matrix-matching employed the calcite reference material WC-1. Differences in ablation yield for dolomite and calcite may compromise the accuracy of the age calculation^56^. However, the application of linear rasters (instead of static spot ablations) minimizes downhole fractionation and thus any related age offsets. We therefore assume that the calculated U–Pb date for the dolomite vein is accurate within the reported uncertainty.

Laser sampling employed ablation of successive linear rasters that were compiled into element, elemental ratio and isotope ratio maps. To reduce the impact of flicker noise and of sequential sampling of different ablation sites during one integration cycle (or mass sweep), we average the original signal over four (most experiments; to five—FA2 main run; Supplementary Table 1) integration cycles. This means that one pixel of the map (= one time-slice) is represented by four (to five) original integration cycles. This results in a pixel width of 60 µm (4 × 500 ms integration = 2 s per time-slice) (FA2 main run: 45 µm; 5 × 300 ms = 1.5 s per time-slice), while the pixel height is determined by the laser spot size of 95 µm.

Characteristic major, minor and trace elements were measured along with U and Pb isotopes. Filtering of the data associated with the pixels in the maps was undertaken by applying specific geochemical criteria to separate pixels from chemically and texturally different domains. However, U–Pb dating of very young carbonate samples (here JH-2 and JH-3) with relatively high µ (^238^U/^204^Pb) by LA-Q-ICP-MS is challenging due to the low concentrations of radiogenic Pb in such samples, and due to the somewhat limited sensitivity of Q-ICP-MS systems. Therefore non-detection of Pb isotopes, in particular ^207^Pb and ^208^Pb, required the use of further selection criteria to filter for Pb isotope signals above the background level. Details on the selection criteria and on the selected pixels (shown in green) are provided with the data tables.

The selected pixels were then pooled into ‘pseudo-analyses’ by using an empirical cumulative distribution function (ECDF) of a channel that is suitable to retrieve the maximum possible spread of the data on isochron diagrams. In this study the ^207^Pb/^235^U ratio was used for pooling. Therefore the Tera-Wasserburg data may include artefacts from pooling (due to low count rates on ^207^Pb) as no outlier correction is applied in Monocle^57^, and, thus, the dates retrieved from 86-TW regressions are preferred.

Uncertainty propagation follows the recommendations of^58^ with the modifications suggested by^59^ and are quoted at 2 s (95% confidence level) The first uncertainty quoted is a session-wide estimate including the data point uncertainty, uncertainty on weighted means of primary reference material ratios and their excess scatter. The second uncertainty additionally includes systematic uncertainties such as the uncertainty on the reference age of WC-1, uncertainty on the ^238^U decay constant and a laboratory-specific long-term reproducibility based on the results of the QC materials.

The general analytical and data processing protocols are described in^60^, while specific details on the analytical method and operating conditions are given in the supplementary Table 1. All U–Pb dates are derived from unanchored model 1 regressions in 86-TW space^61^, which is a modification (^208^Pb_common_/^206^Pb_total_ *versus* ^238^U/^206^Pb_total_) of the Tera-Wasserburg concordia.

## Supplementary Information

Supplementary Information.

Supplementary Table 1.

Supplementary Table 2.

Supplementary Table 3.

## Data Availability

Sample and data acquisition locations, U–Pb and geochemical data tables and analytical technique are provided in the Supplemental Material.
