# Supplementary material for: The influence of Cenozoic Eurasia-Arabia convergence on the Southeast Arabian Foreland Basin: new geochronological and geochemical constraints from syn-kinematic carbonate mineralization
Source: Sci Rep. 2023 Mar 16;13:4387. doi: 10.1038/s41598-023-31611-x (PMC10020455; doi:10.1038/s41598-023-31611-x)
Supplement: Supplementary file 1 — Supplementary Information. [file 41598_2023_31611_MOESM1_ESM.pdf]

The influence of Cenozoic Eurasia-Arabia convergence on the Southeast Arabian Foreland Basin: new geochronological and geochemical constraints from syn-kinematic carbonate mineralization

Francesco Arboit[a,b*], Kerstin Drost[c], Alessandro Decarlis[a,b], David Chew[c], Dominik Hennhoefer[a], Andrea Ceriani[a,b]

[a]Department of Earth Sciences, Khalifa University of Science and Technology, Abu Dhabi, UAE
[b]Research and Innovation Center on CO_2 and H_2 (RICH), Khalifa University of Science and Technology, Abu Dhabi, UAE
[c]Department of Geology, School of Natural Sciences, Trinity College Dublin, Dublin 2, Ireland

Triassic to Cenozoic stratigraphy of Oman and the United Arab Emirates

During the early Mesozoic, the UAE region was located in an equatorial setting as part of a large carbonate platform on the rifted southern continental margin of the Neo-Tethys Ocean. The early stages of rifting were in the Mid-Permian (Glennie et al. 1973; Béchennec et al., 1990), as is evidenced by the Jabal Qamar exotic in the Dibba Zone, and Mid-Permian syn-rift (Bih Formation) in the northern Omani mountains, while the later stages were in the Late Triassic – Early Jurassic (Glennie et al., 1973; Searle et al., 1983; Searle, 1988; Robertson et al., 1990; Béchennec et al., 1990). The early rift stage was dominated by continental rifting and block-faulting with more localised within-plate off-axis volcanism, while the later stage was characterised by volcanism, tilted fault blocks, half-grabens and growth on the main border faults, leading to break-up (Robertson and Searle, 1990). Further, geochemical analyses of volcanic rocks in Saih Hatat in the central Oman mountains suggest that oceanic crust was formed in the Late Triassic (Searle, 2007). By the end of the mid-Cretaceous, the region subjected to compressional deformation, which involved the emplacement of compressive deformations from NE to SW onto the Neo-Tethyan rifted continental margin (Glennie et al., 1973; Lippard et al., 1986; Searle, 1988). The embricated sheets involve the Sumeini Complex, shelf-edge and slope-carbonate sediments; the Hawasina Complex, comprising distal-slope and deep-sea Neo-Tethyan sediments; Haybi Complex, comprising Permian to Cenomanian exotic limestones (Oman exotics), volcanics (Haybi volcanics), mélanges, sub-ophiolitic metamorphic rocks, and the Semail Ophiolite complex, a massive ‘slab’ (up-to 8–15 km thick) of oceanic crust and mantle of Cenomanian – Turonian age, which formed above an east-dipping intra-oceanic subduction zone (Searle and Cox, 1999). The compressive deformation ended c. by 70 Ma (Early Maastrichtian) (Searle, 2007). The obduction of the Semail Ophiolite and its associated thrusts and folds loaded and then flexed the pre-existing underlying rift margin sediments. The flexure formed the UAE foreland basin and flanking bulge, which together migrated westward in front of the advancing ophiolite load. The Upper Cretaceous foreland basin was infilled by an up to 4.3 km thick Santonian − Campanian deep-marine mudstones of the Fiqa and Juwaiza formations, which rapidly increase in thickness towards the northeast (Glennie et al., 1973). This Upper Cretaceous foreland sequence has been later overlain by the Upper Maastrichtian to Palaeogene conglomerates and shallow-marine limestone of the Qahlah and Simsima formations (Glennie et al., 1973; Lippard et al., 1986). The margin remained stable until post-Middle Eocene time through the deposition of the transgressive Umm Er Radhuma, Rus, Dammam, Asmari and Fars formations (Ali and Watts, 2009).

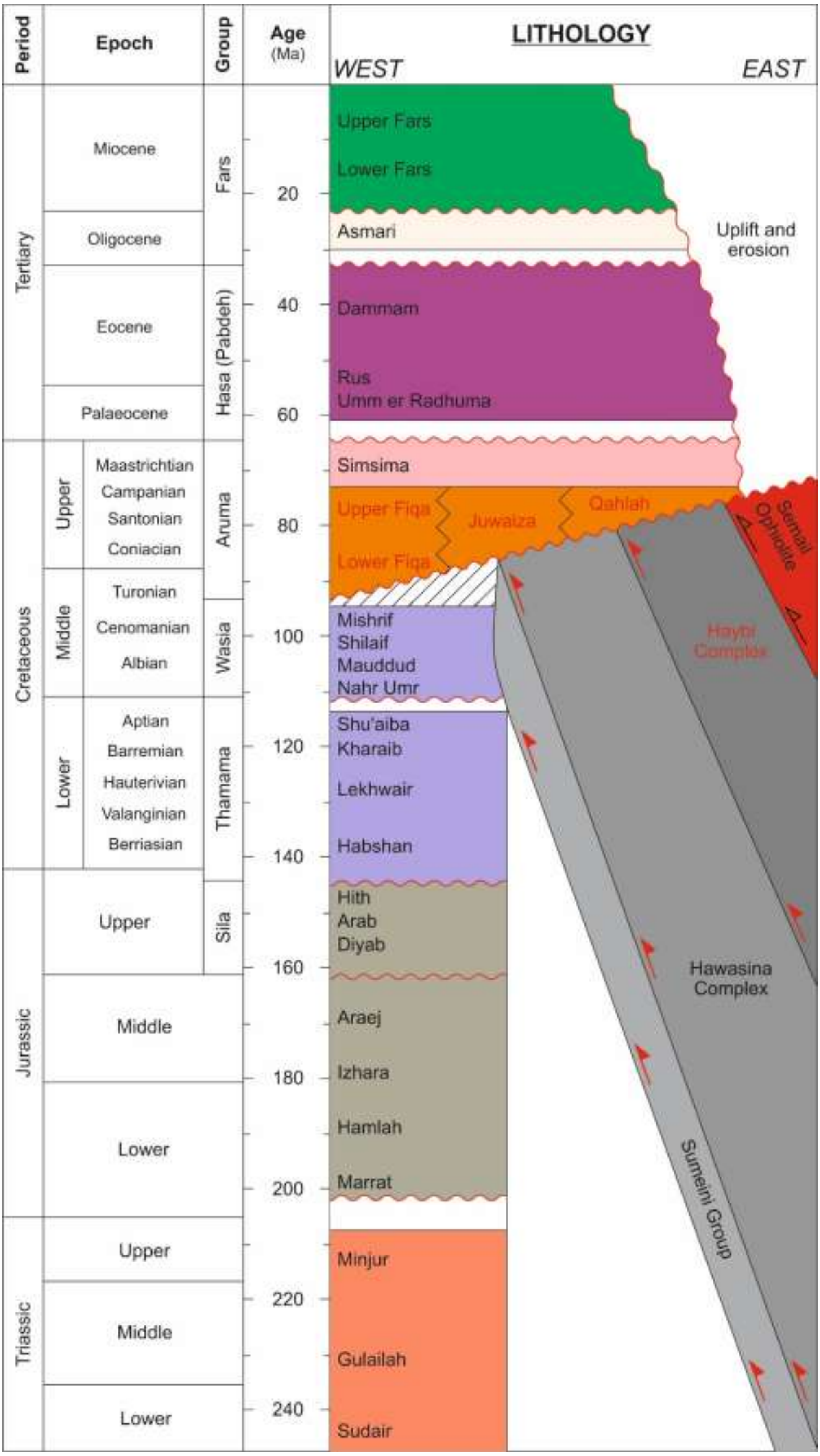

Figure 1. Triassic to Miocene stratigraphy of Oman and the UAE. Modified from Ali and Watts (2009).

Reference list

Ali, M., Watts, A.B. Subsidence history, gravity anomalies and flexure of the United Arab Emirates (UAE) foreland basin. *GeoAr*. **14**(2), 17-44 (2009).

Béchennec, F., J. LeMetour, D. Rabu, C.H. Bourdillon-de-Grissac, P. DeWever, M. Beurrier and M. Villey. The Hawasina nappes: Stratigraphy, palaeogeography and structural evolution of a fragment of the south Tethyan passive continental margin. In A.F.H. Robertson, M.P. Searle and A.C. Ries (Eds.), The Geology and Tectonics of the Oman Region. *Geol. Soc. of London, Spec. Pub*. **49**, 213-223 (1990).

Glennie, K.W., M.G.A. Boeuf, M.W. Hughes Clark, M. Moody-Stuart, M.F. Pilaar and B.M. Reinhardt. Late Cretaceous nappes in the Oman Mountains and their geological evolution. *AAPG Bull*. **57**, 5-27 (1973).

Lippard, S.J., A.W. Shelton and I.G. Gass. The Ophiolite of Northern Oman. *Geol. Soc. of London Memoir*, **11**, 1-178 (1986).

Robertson, A.H.F., C.D. Blome, D.W.J. Cooper, A.E.S. Kemp and M.P. Searle. Evolution of the Arabian continental margin in the Dibba Zone, Northern Oman Mountains. In A.F.H. Robertson, M.P. Searle and A.C. Ries (Eds.), The Geology and Tectonics of the Oman Region. *Geol. Soc. of London, Spec. Pub*. **49**, 251-284 (1990).

Robertson, A.H.F. and M.P. Searle. The northern Oman Tethyan continental margin. In A.F.H. Robertson, M.P. Searle and A.C. Ries (Eds.), The Geology and Tectonics of the Oman Region. *Geol. Soc. of London, Spec. Pub*. **49**, 3-25 (1990).

Searle, M.P. Structure of the Musandam culmination (Sultanate of Oman and United Arab Emirates) and the Straits of Hormuz syntaxis. *J. of the Geol. Soc. of London*, **145**, 831-845 (1988).

Searle, M.P. Structural geometry, style and timing of deformation in the Hawasina Window, Al Jabal al Akhdar and Saih Hatat culminations, Oman Mountains. *GeoAr.* **12**(2), 99-130 (2007).

Searle, M.P., N.P. James, T.J. Calon and J.D. Smewing. Sedimentological and structural evolution of the Arabian continental margin in the Musandam Mountains and Dibba zone, United Arab Emirates. *Geol. Soc. of Am. Bull.* **94**, 1381-1400 (1983).

Searle, M.P. and J. Cox. Subduction zone metamorphism during formation and emplacement of the Semail ophiolite in the Oman Mountains. *Gel. Mag.* **139**, 241-255 (1999).

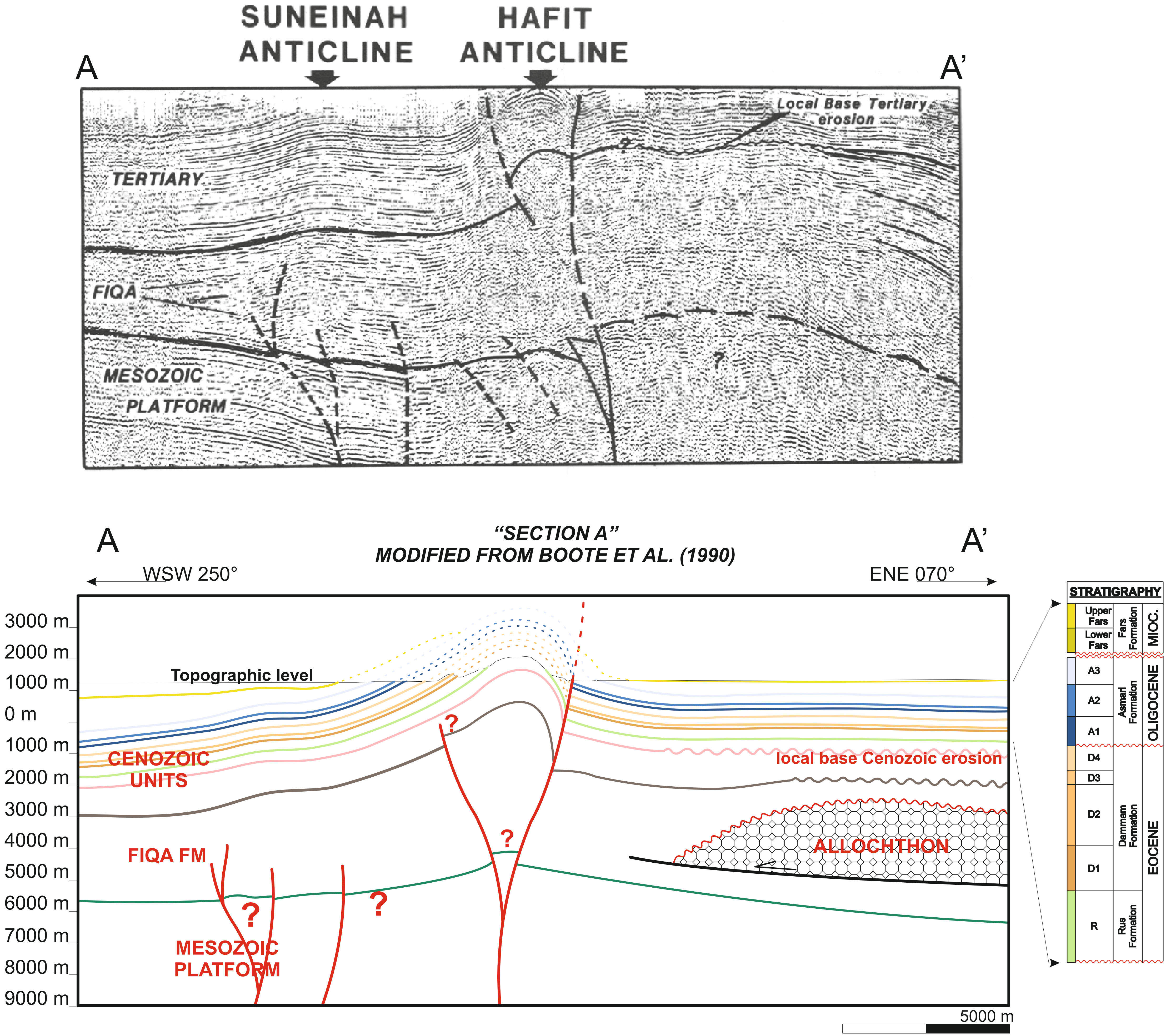

Boote, D.R.D., Mou, D., Waite, R.I. Structural evolution of the Suneinah foreland, central Oman Mountains. *Geol. Soc. Lond. Sp. Publ.* **49**(1), 397-418 (1990).

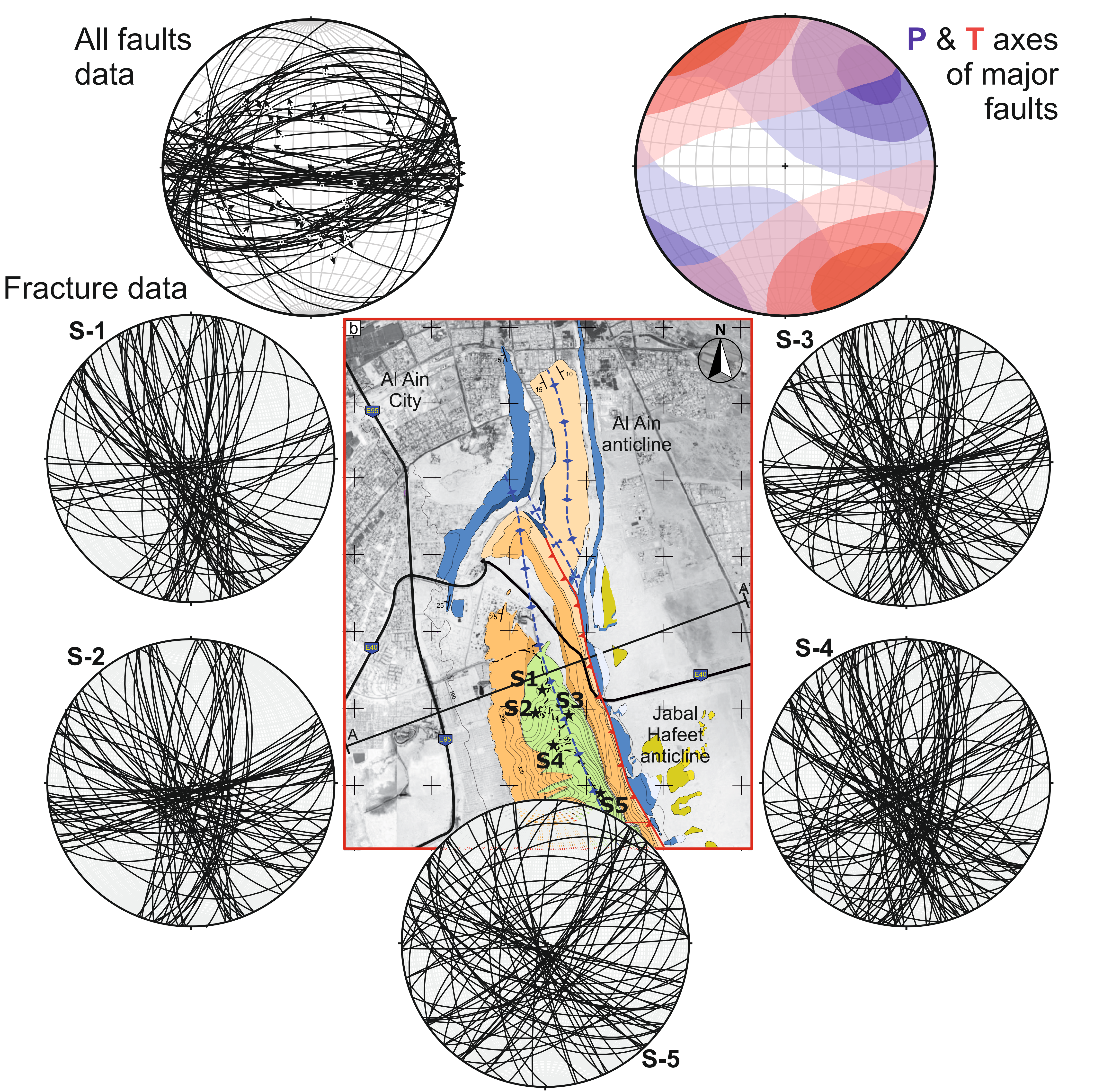
All faults data
P & T axes of major faults
Fracture data
S-1
S-2
S-3
S-4
S-5
b
N
Al Ain City
Al Ain anticline
Jabal Hafeet anticline
E95
E40
A
A'
S1
S2
S3
S4
S5

PHOTOGRAPHIC REPORT JABAL HAFEET FIELDWORK

Photo 1

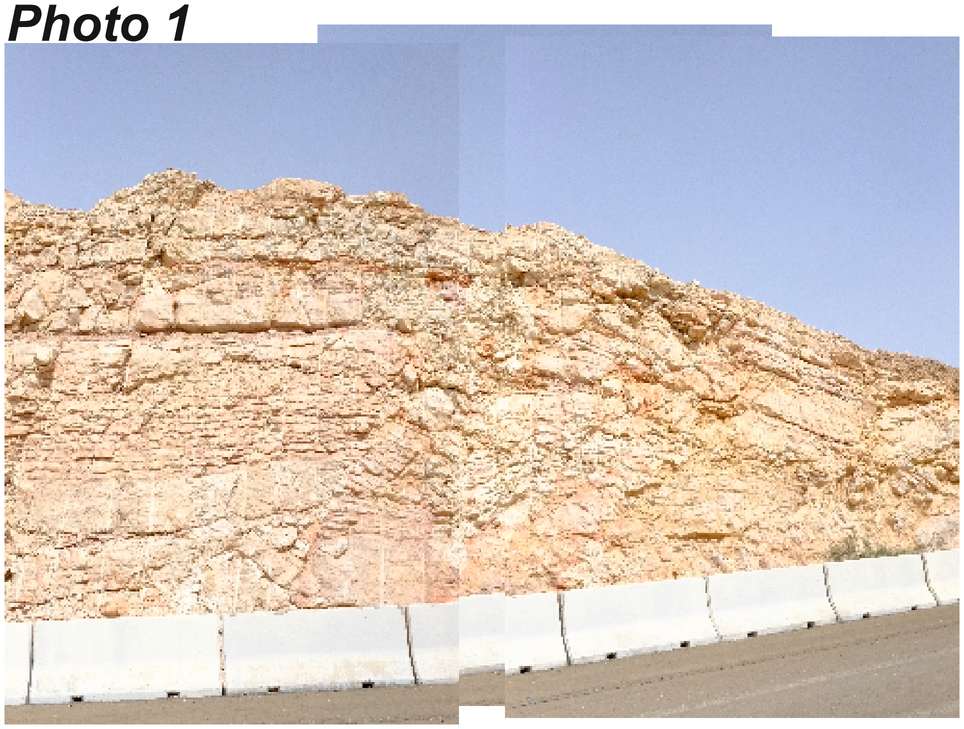

Photo 1:

Panoramic collage of an imbricated compressive fault system.
coo: 24.091159N, 55.764649E

Photo 2

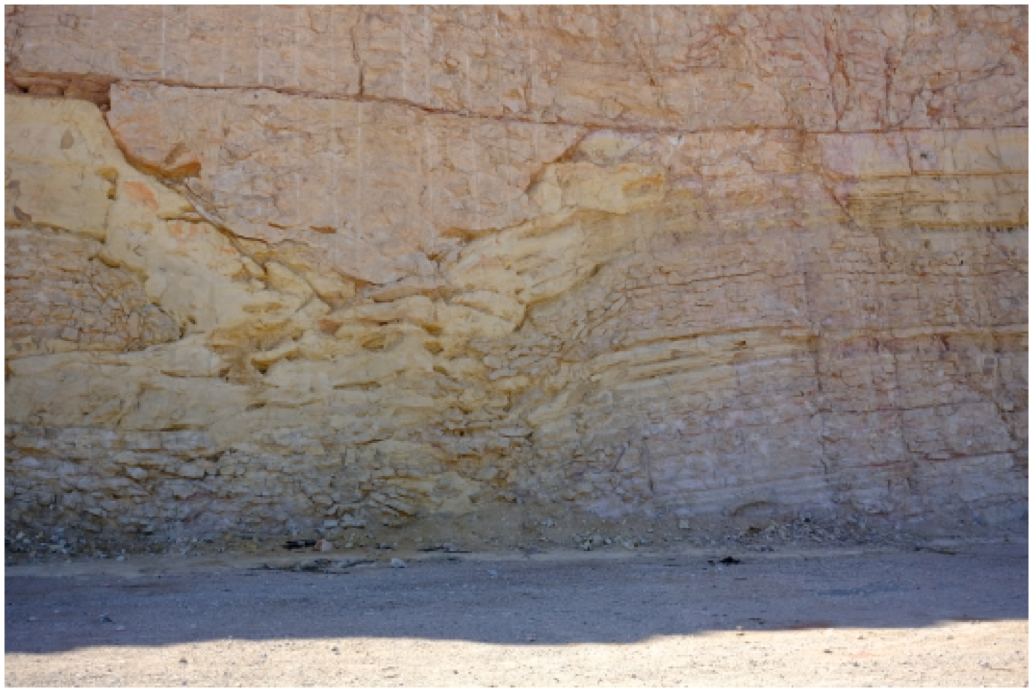

Photo 2:

Collapsed silt channel within shallow marine facies of the Rus Formation.
coo: 24.092477N, 55.765531E

Photo 3

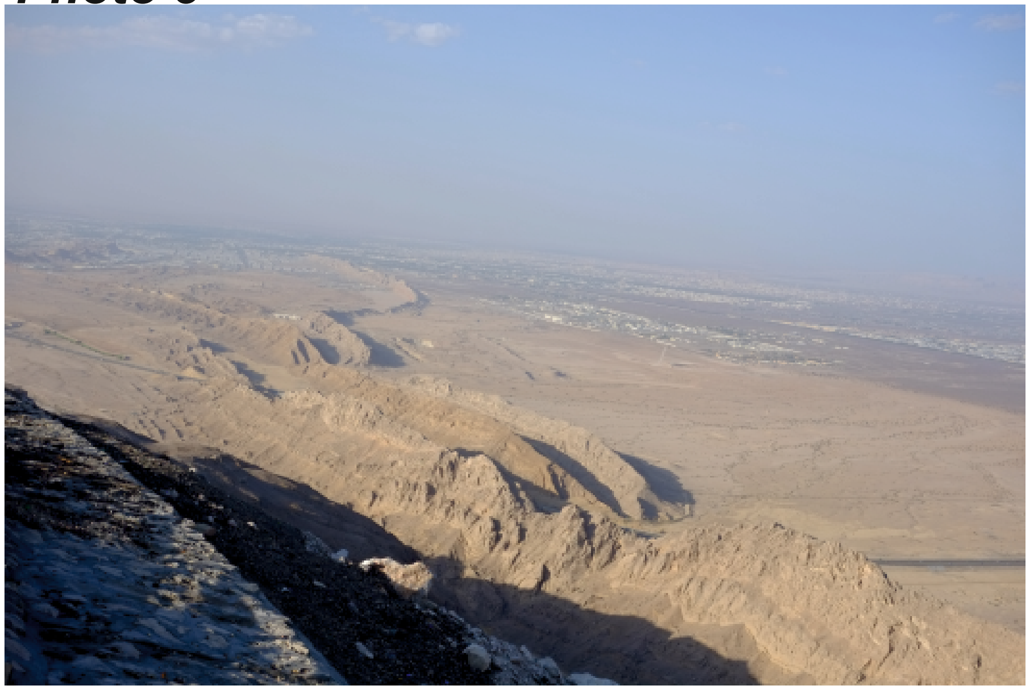

Photo 3:

Top view of the Rwaidhat syncline (on the left) and of the Al Ain anticline (on the right) from the eastern limb of the Jabal Hafeet anticline.
The photo looks north.

PHOTOGRAPHIC REPORT JABAL HAFEET FIELDWORK

Photo 4

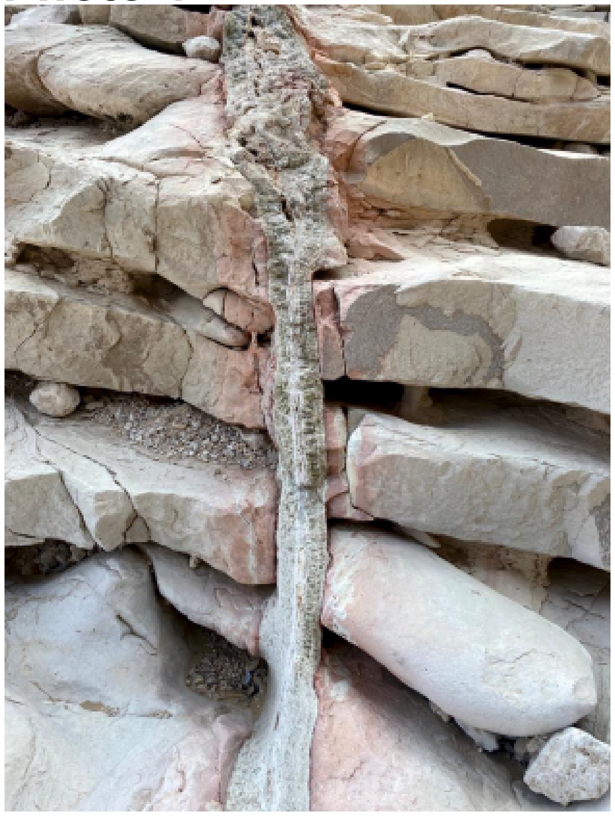

Photo 4:
Strike-slip shear plane reactivated as extensional fault filled with green to brown coloured fibrous calcite.
Location Sample JH-3.
coo: 24.08743N, 55.76599E

Photo 5

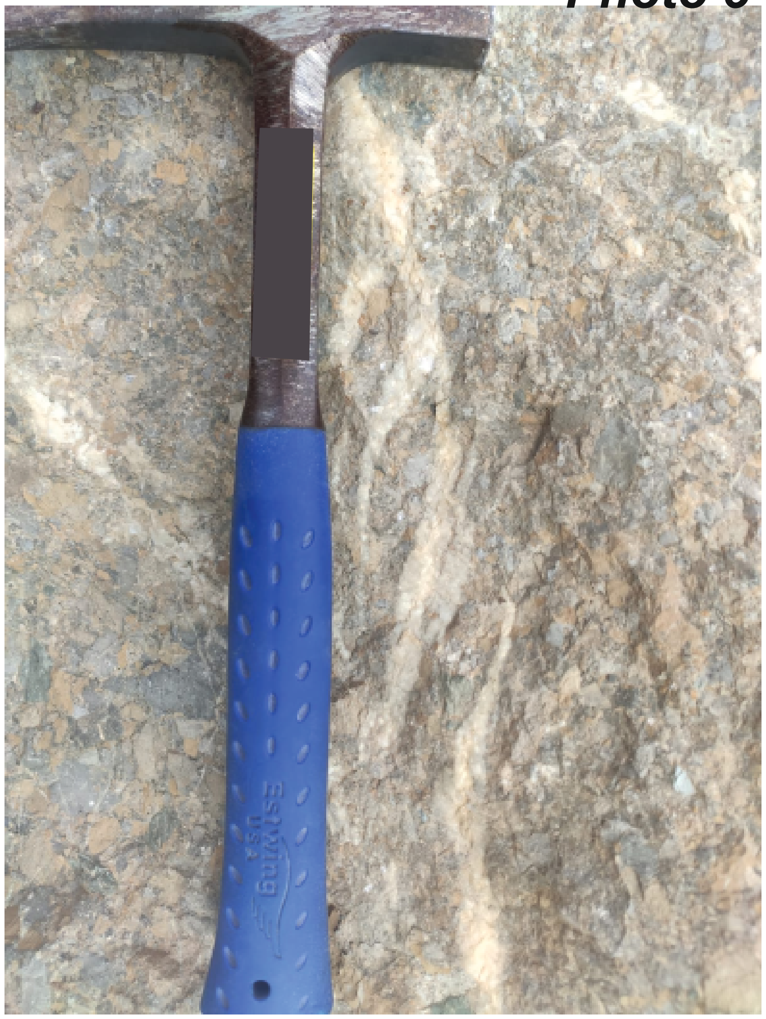

Photo 5:
En echelon strike-slip veins of dolomite cement.
coo: 24.097201N, 55.762294E

Photo 6

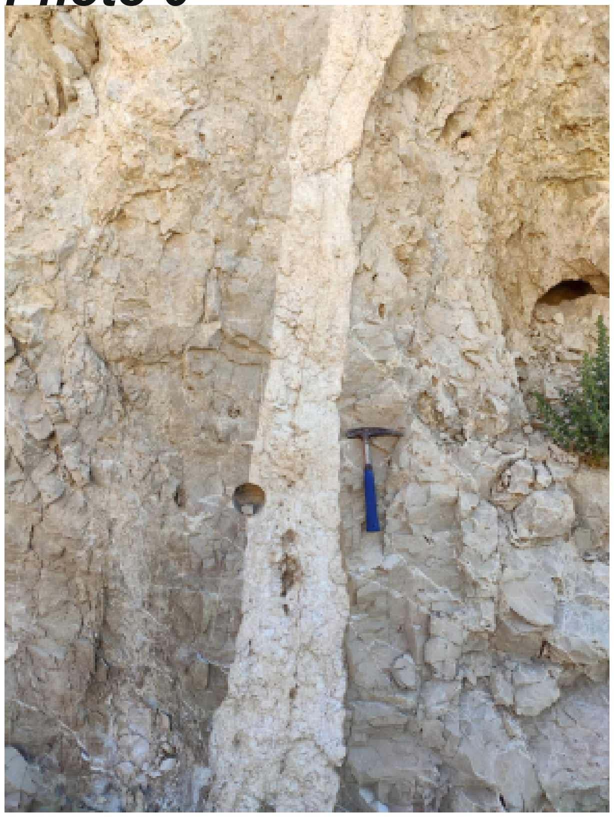

Photo 6:
Decimetric E-W striking strike-slip shear plane filled with centimetric crystals of equant drusy calcite.
coo: 24.094282N, 55.759800E

PHOTOGRAPHIC REPORT JABAL HAFEET FIELDWORK

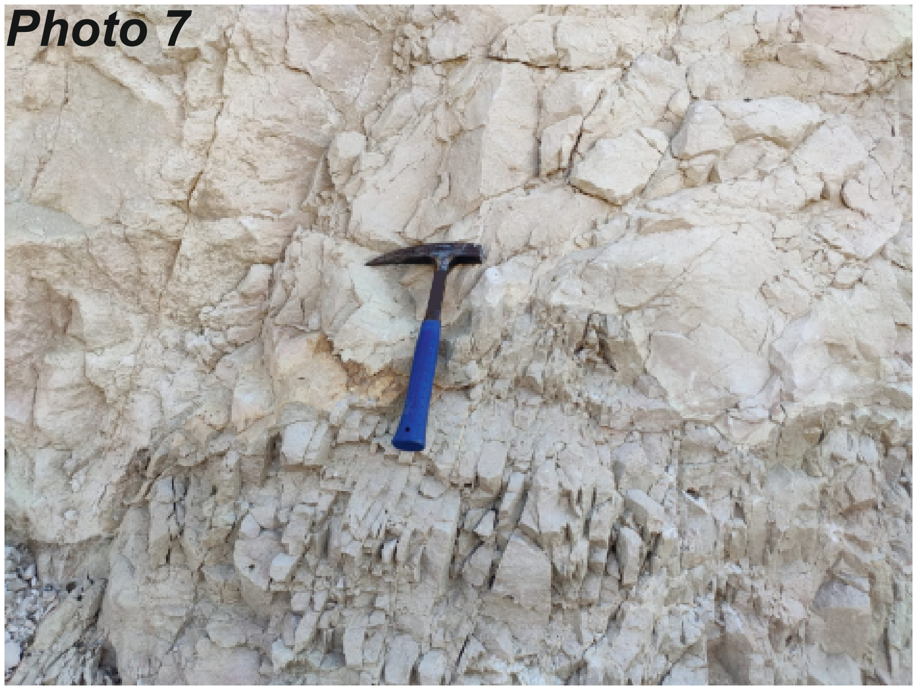

Photo 7:
Picture showing the different mechanical behaviour of calcite (clear white rocks on top of the picture) and dolomite rocks (clear gray rocks, bottom of the photo) subjected to the same stress regimes.
coo: 24.097362N, 55.759343E

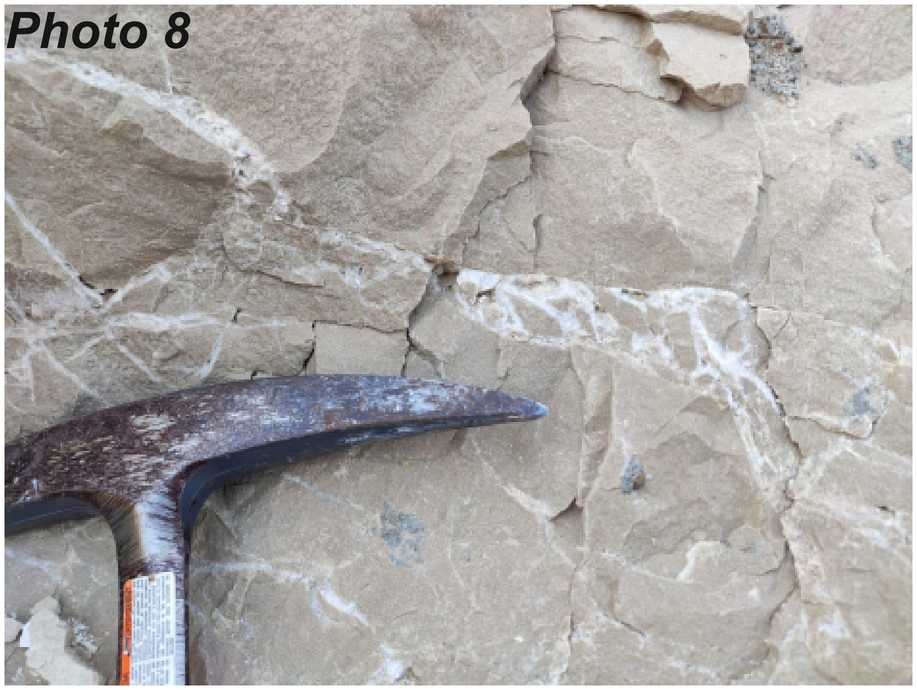

Photo 8:
Strike-slip brecciated shear zone filled with calcite cement in dolomite host rock.
coo: 24.096070N, 55.758754E

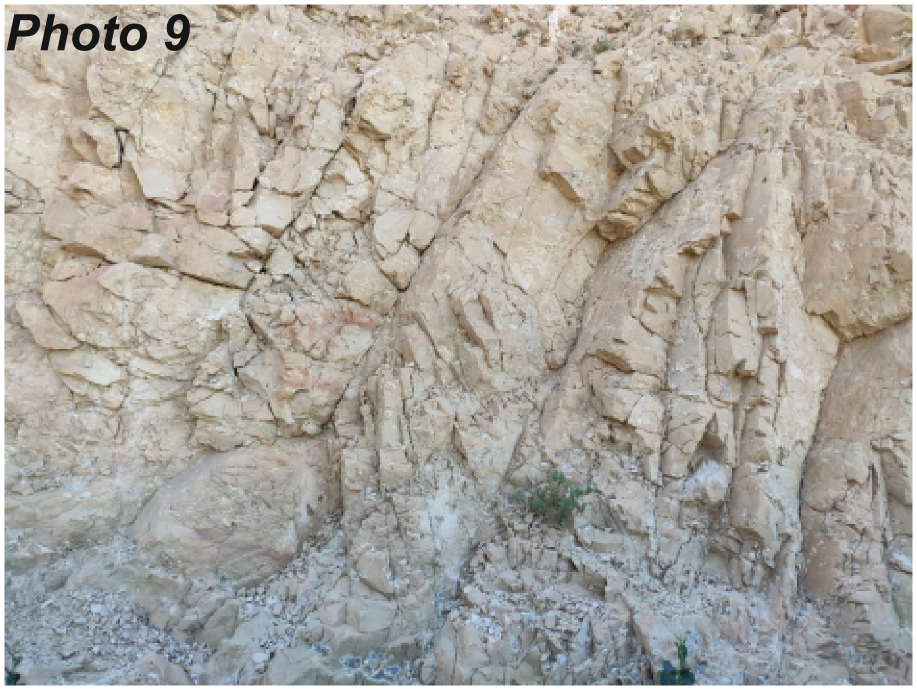

Photo 9:
Damage zone of a E-W strike-lip shear zone deforming the carbonates of the Rus Formation.
coo: 24.096070N, 55.758754E

PHOTOGRAPHIC REPORT JABAL HAFEET FIELDWORK

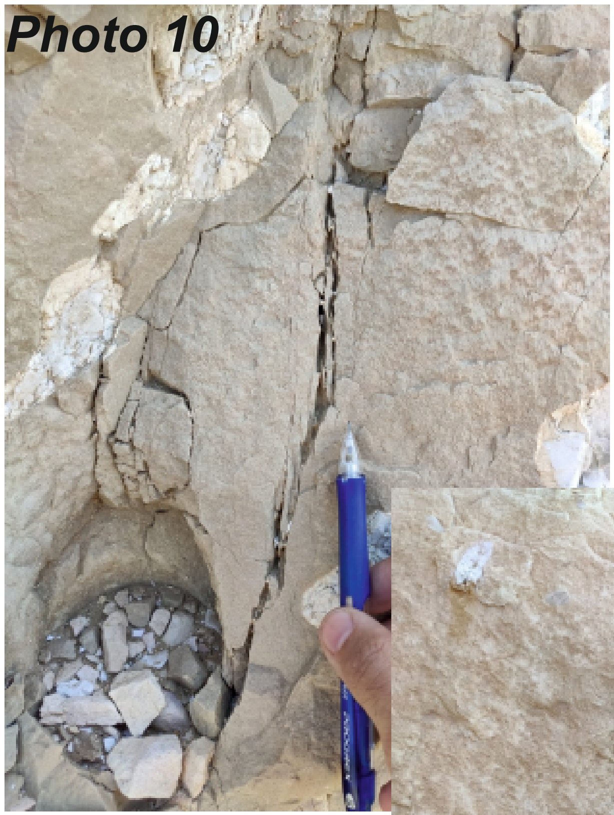

Photo 10:
Picture showing an E-W striking open strike-slip shear zone within the dolomite rocks of the Rus Formation.

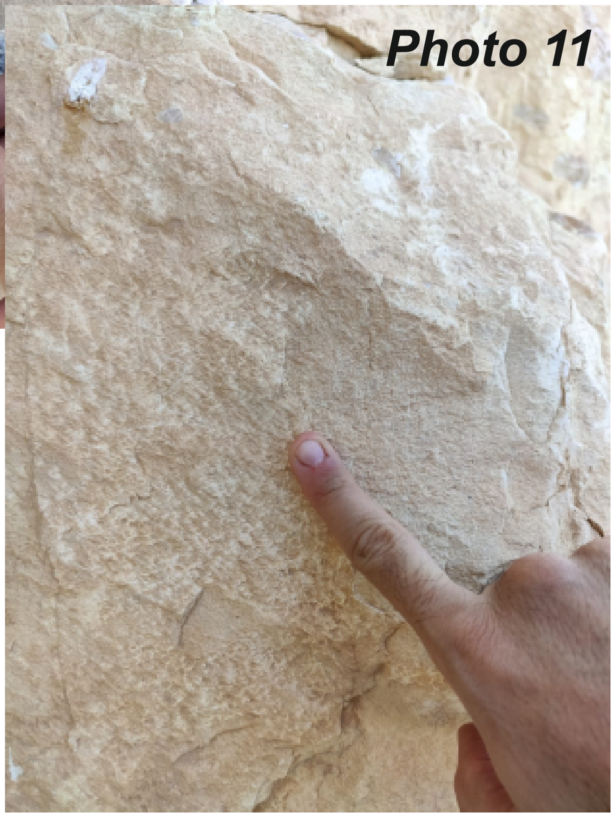

Photo 11:
Transpressional shear plane with stylolites showing the strike-slip kinematics of the fault .

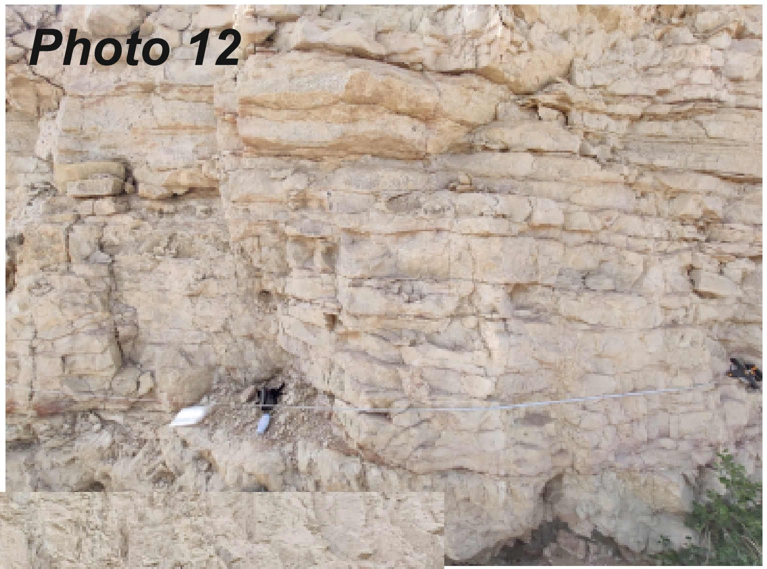

Photo12:
Location of S1.

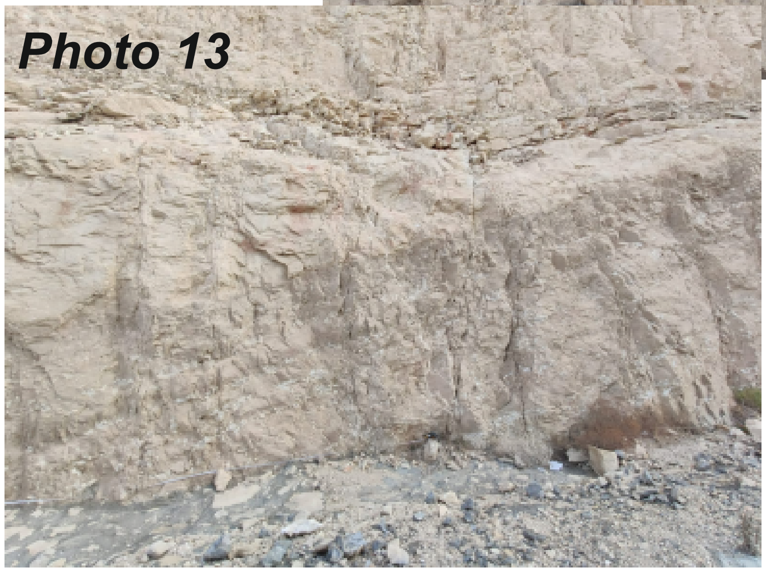

Photo 13:
Location of S2.

PHOTOGRAPHIC REPORT JABAL HAFEET FIELDWORK

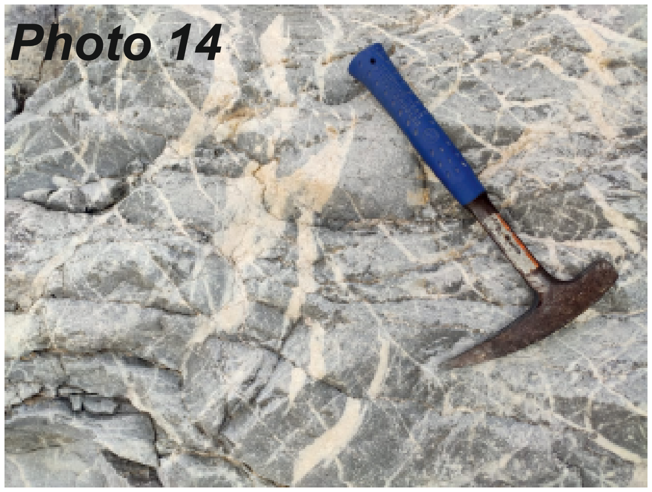

Photo 14:
Photo showing multiple generations of shear events on dolomitic layers of the Rus Formation.
coo:24.098846N, 55.763193E

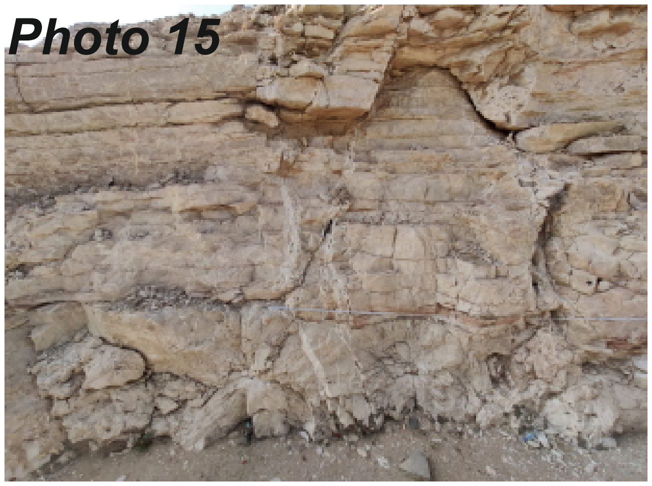

Photo 15:
Location of S3.

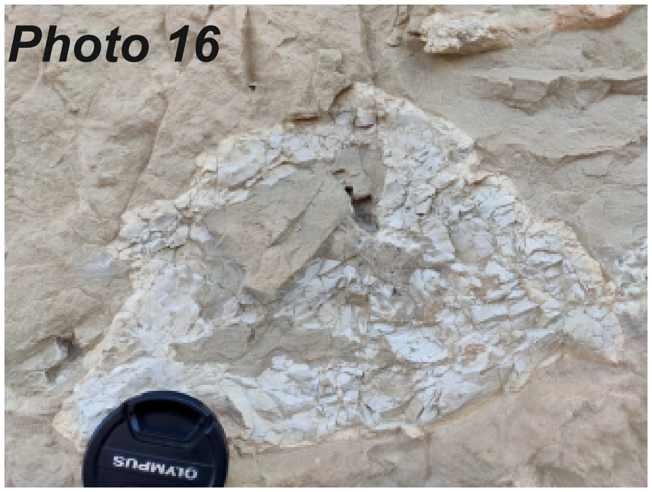

Photo 16:
Photo showing the dissolution and mobilitization of the silica, from the original chert nodules into nodules with brecciated dolomitic host rock. This paragenetic sequence proves the silica mobilitization event happened during or after the dolomitization of the Rus Formation.
Similar to the Eocene Corones carbonates of the Spanish Pyrenees.
coo: 24.096792N, 55.758941E

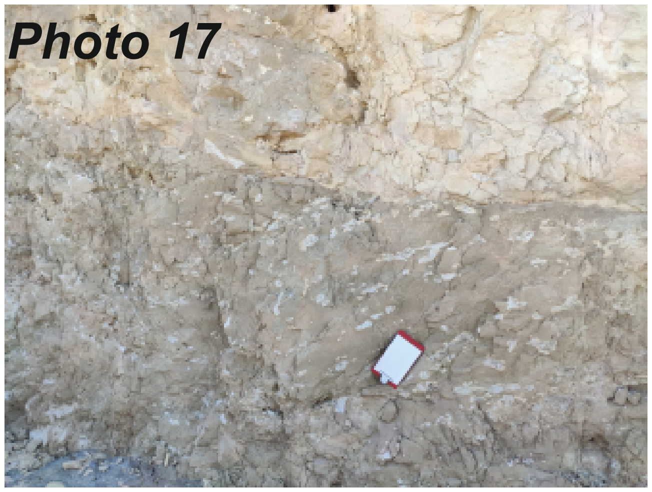

Photo 17:
Dolomite (bottom gray rocks) in tectonic contact with the biogenic limestone (top white rocks) of the Rus Formation.

PHOTOGRAPHIC REPORT JABAL HAFEET FIELDWORK

Photo 18

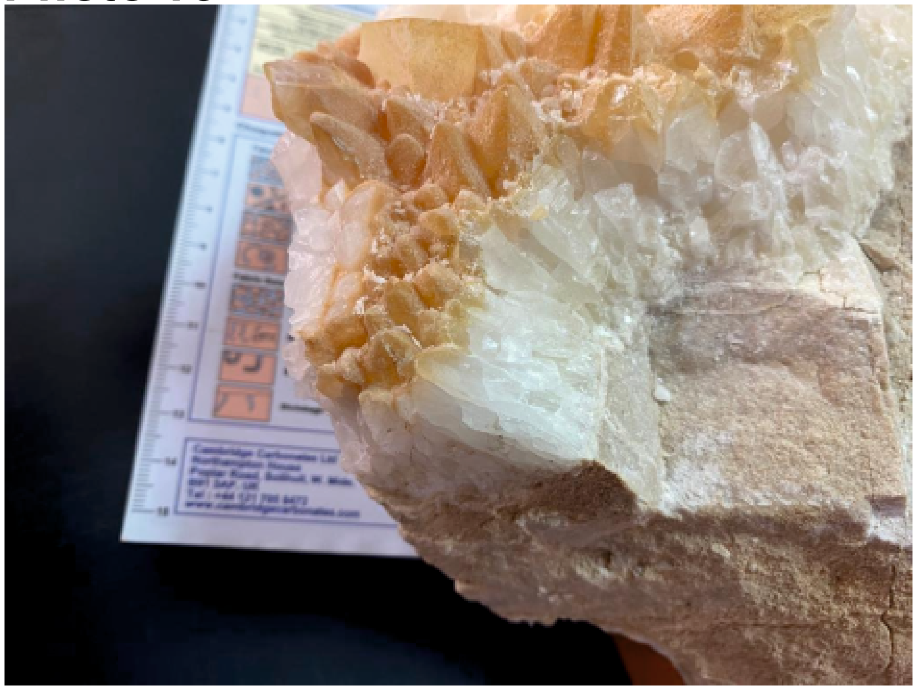

Photo 18:
Photo of hand specimen of sample JH-2 characterized by centimetric dog-tooth calcite crystals covered by a layer of μm-sized crystals of hematite.
coo: 24.08675N, 55.76291E

Photo 19

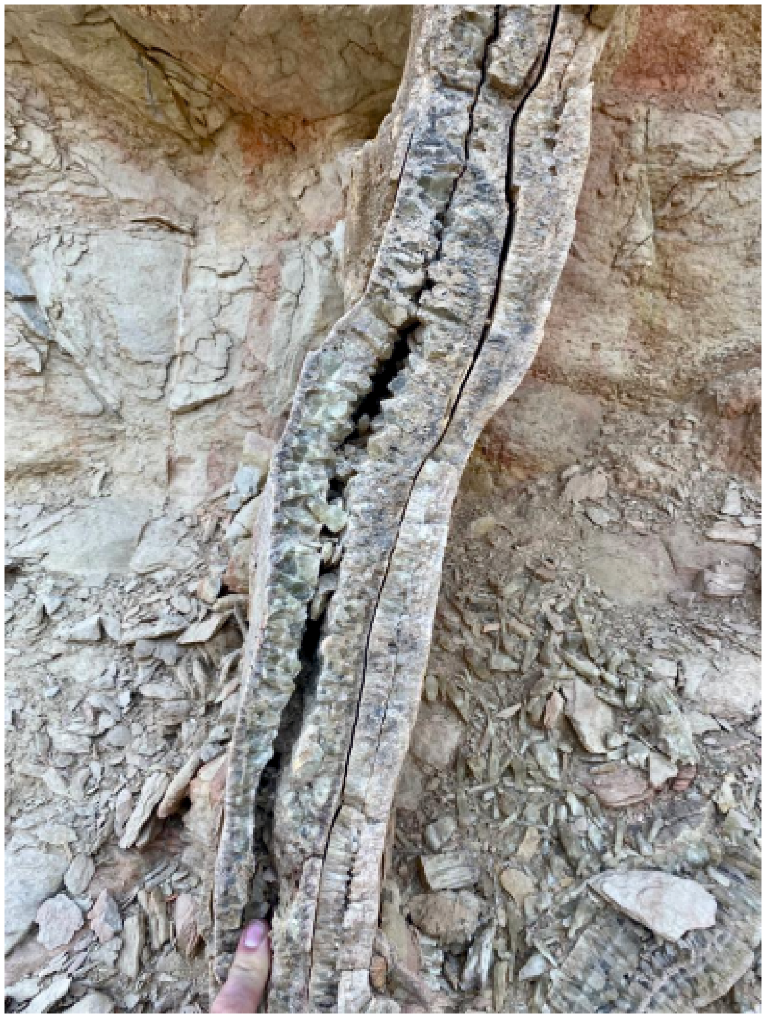

Photo 19:
ENE-WSW strike-slip shear plane reactivated as mode-I tensile fracture during late stages of uplift of the anticline.
Vein filled with fibrous gray-green calcite crystals.
coo: 24.065649N, 55.776987E

Photo 20

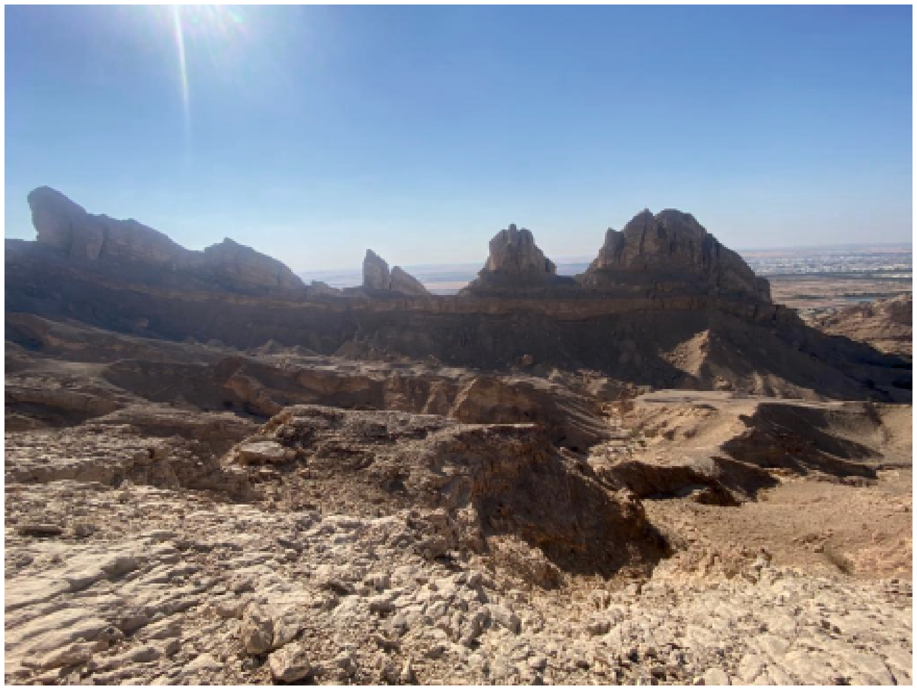

Photo 20:
Panoramic view of the back-limb of the Jabal Hafeet anticline (looking ESE) showing the contact between the Rus Formation and the overlaying Dammam Formation.
Photo taken standing on the Rus Formation.

PHOTOGRAPHIC REPORT JABAL HAFEET FIELDWORK

Photo 21

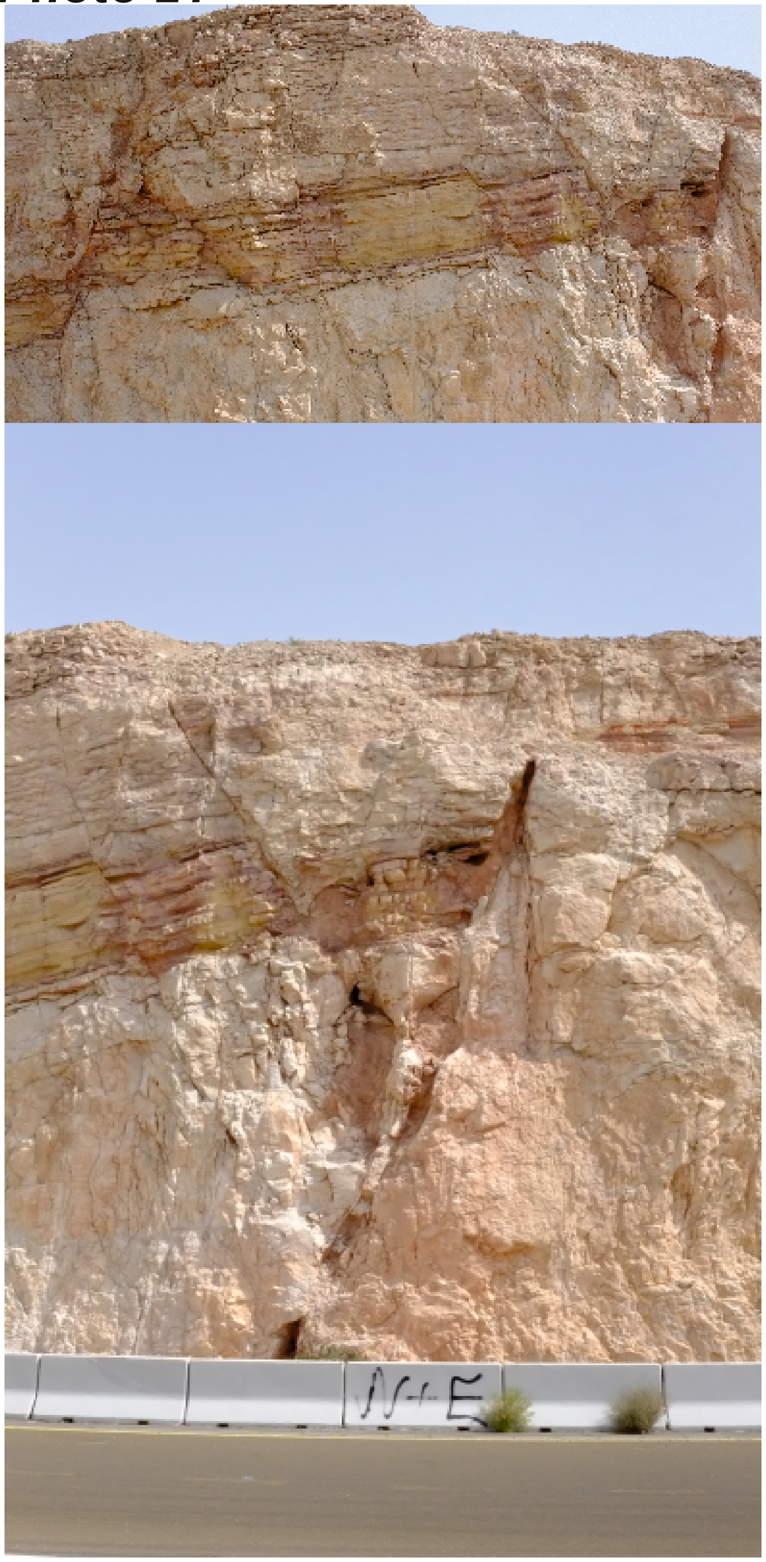

Photo 21:
ENE-WSW striking normal faults that deformed the rocks of the Rus Formation mostly in the hinge zone of the anticline during the gravitational collapse stage due to the doming of the anticline.
Steep dipping (dip: 80º±15º) strike-slip faults reactivated as extensional shear planes.

Photos collected showing the deformation clastic channels into the shallow marine facies of the Rus Formation.
coo: 24.085503N, 55.765899E

Photo 22

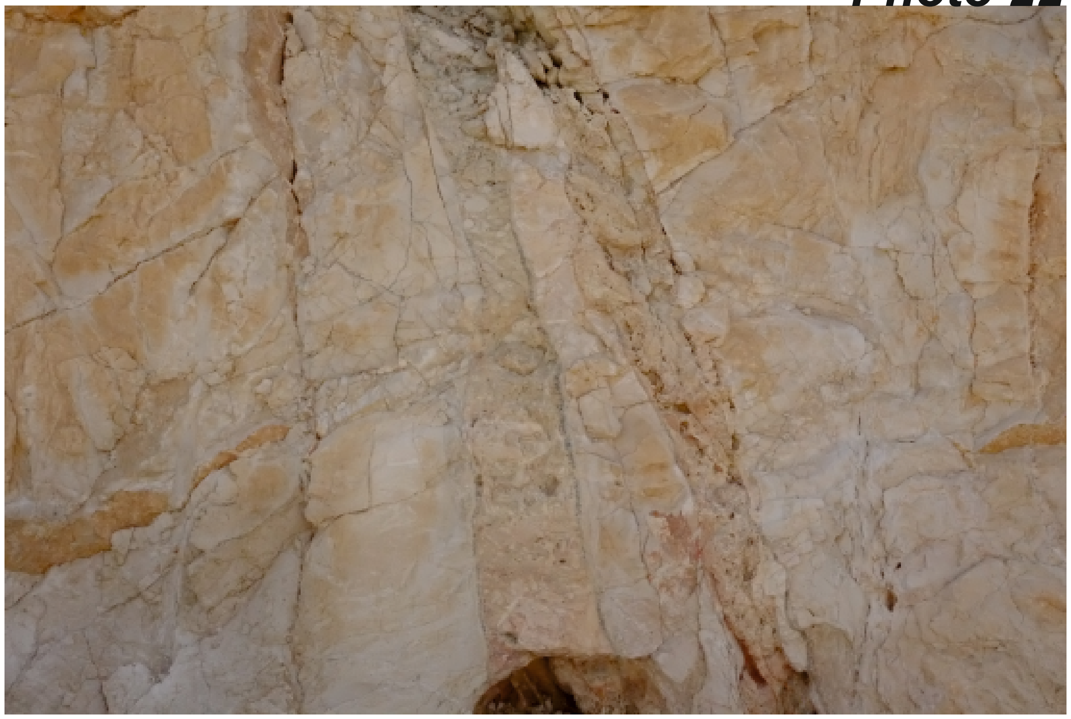

Photo 22:
Conjugate strike-slip shear planes cross cutting compressive faults.

Same location as Sample JH-1.
coo: 24.09188N, 55.76657E
