# Supplementary material for: The influence of Cenozoic Eurasia-Arabia convergence on the Southeast Arabian Foreland Basin: new geochronological and geochemical constraints from syn-kinematic carbonate mineralization
Source: Sci Rep. 2023 Mar 16;13:4387. doi: 10.1038/s41598-023-31611-x (PMC10020455; doi:10.1038/s41598-023-31611-x)
Supplement: Supplementary file 2 — Supplementary Table 1. [file 41598_2023_31611_MOESM2_ESM.pdf]

The influence of Cenozoic Eurasia-Arabia convergence on the Southeast Arabian Foreland Basin: new geochronological and geochemical constraints from syn-kinematic carbonate mineralization

Francesco Arboit[a,b*], Kerstin Drost[c], Alessandro Decarlis[a,b], David Chew[c], Dominik Hennhoefer[a], Andrea Ceriani[a,b]

[a]Department of Earth Sciences, Khalifa University of Science and Technology, Abu Dhabi, UAE
[b]Research and Innovation Center on CO_2 and H_2 (RICH), Khalifa University of Science and Technology, Abu Dhabi, UAE
[c]Department of Geology, School of Natural Sciences, Trinity College Dublin, Dublin 2, Ireland

Data reporting Template (Information) for LA-ICP-MS U-Th-Pb data

**Laboratory & Sample Preparation**	
Laboratory name	Dept of Earth Science, Trinity College Dublin
Sample type/mineral	calcite and dolomite veins
Sample preparation	1 inch resin mount, 1μm polish to finish
Imaging	high resolution scan of sample
**Laser ablation system**	
Make, Model & type	Teledyne/PhotonMachines Analyte Excite, 193nm, Excimer
Ablation cell & volume	HelEx II Active 2-volume cell; 100mm × 100mm sample area
Laser wavelength (nm)	193nm
Pulse width (ns)	<4ns
Fluence ($J.cm^{-2}$)	2.0 J/cm^2
Repetition rate (Hz)	50 Hz
Spot size (um)	95 μm
Sampling mode / pattern	linear raster, 1 pass, 30 μm/sec scan speed
Carrier gas	100% He in the cell, Ar make-up gas and N2 (optimized daily: 6.5 to 11.5 mL/min) combined via a y-connector and added to He+aerosol at tangential inlet of ARIS adaptor
Ablation duration (secs), map dimensions (width x height in μm), map resolution (pixel width x height, μm)	FA1 test<br>map: 4300 x 1900μm (143s x 20 linear rasters, c. 48min of total signal)<br>FA2 test<br>map: 3090 x 1900μm (103s x 20 linear rasters, c. 34min of total signal)<br>FA1 repeat<br>map: 15840 x 1900μm (528s x 20 linear rasters, c. 176min of total signal)

	FA3<br>map: 2760 x 6745µm (92s x 71 linear rasters, c. 109min of total signal)<br>FA3 repeat<br>top map: 6000 x 1900µm (200s x 20 linear rasters, c. 67min of total signal)<br>bottom map: 6000 x 1900µm (200s x 20 linear rasters, c. 67min of total signal)<br>FA2 main run<br>top map: 4860 x 1900µm (162s x 20 linear rasters, c. 54min of total signal)<br>bottom map: 6780 x 1330µm (226s x 14 linear rasters, c. 53min of total signal)
Cell carrier gas flow (l/min)	0.4 l/min
**ICP-MS Instrument**	
Make, Model & type	Agilent 7900 quadrupole ICP-MS
Sample introduction	Ablation aerosol via ARIS
RF power (W)	1550W
Make-up gas flow (l/min)	optimized daily: 0.60 to 0.75 L/min Ar
Detection system	Dual-mode discrete dynode electron multiplier
Masses measured [Integration time per peak in ms]	FA2 main run: 25 [5], 43 [5], 51 [2], 55 [2], 57 [4], 63 [2], 71 [2], 85 [2], 88 [2], 137 [2], 140 [2], 202 [1], 204 [1], 206 [40], 207 [80], 208 [40], 232 [30], 238 [40]<br>all other experiments: 25 [1.5], 43 [3], 51 [2], 55 [1.5], 57 [1.5], 63 [2], 66 [2], 71 [2], 85 [2], 88 [1.5], 137 [2], 140 [2], 202 [1], 204 [1], 206 [80], 207 [200], 208 [80], 232 [15], 238 [60]
Total integration time per reading (secs)	FA2 main run: 300 ms / 1500 ms after averaging<br>all other experiments: 500 ms / 2000 ms after averaging
Sensitvity / Efficiency (%, element)	0.02% U
IC Dead time (ns)	38ns
**Data Processing**	
Gas blank	≥15s on-peak zero subtracted
Calibration strategy	NIST614 as primary reference material, WC-1 carbonate standard for matrix matching of $^{206}Pb/^{238}U$, Duff Brown Tank lacustrine limestone as well as ASH-15-D-K and ASH-15-C speleothem for QC
Reference Material info	NIST614 (concentration data Jochum et al., 2011; Pb isotopes Woodhead and Hergt, 2001)<br>WC-1 (Roberts et al., 2017)<br>DBT (Hill et al., 2016)<br>ASH-15-D-K (Nuriel et al., 2021)<br>ASH-15-C (Vaks et al., 2013)
Data processing package used / Correction for LIEF	Iolite V3.6 (Paton et al., 2011) & Monocle (Petrus et al., 2017) & in-house spreadsheet; no LIEF correction for linear rasters
Normalisation and age calculation	standard bracketing; Iolite Data Reduction Scheme VizualAge_UcomPbine (Chew et al. 2014; based on U-Pb Geochronology

	DRS of Paton et al., 2010 and VizualAge DRS of Petrus and Kamber, 2012) is used to correct for down hole fractionation and drift and to normalize to primary reference material. Downhole fractionation for linear rasters is modelled using a linear correction (y=a+bx) with zero slope (b=0). U/Pb ages and initial Pb compositions are calculated using Isoplot v4.15 (Ludwig, 2012).
Common-Pb correction, composition and uncertainty	Samples and QC materials: Unanchored regression in Tera-Wasserburg, isochron and 86TW plots, respectively. All model 1.<br>WC-1: Anchored regression in TW using an initial $^{207}Pb/^{206}Pb$ of 0.85±0.04 (Roberts et al., 2017) to receive a non-matrix-matched lower intercept age, the corresponding ratio of which is used to calculate the matrix-dependent factor for correction of $^{206}Pb/^{238}U$ ratios of QC and unknowns
Uncertainty level & propagation	Ratios and ages are quoted at 2s. Uncertainty propagation was carried out according to the recommendations of Horstwood et al. (2016) and Roberts et al. (2020). The first uncertainty quoted is a session wide estimate including the data point uncertainty, uncertainty on weighted means of primary reference material ratios and their excess scatter. The second uncertainty quoted additionally includes systematic uncertainties such as the uncertainty on the reference age of WC-1, uncertainty on the 238U decay constant and a laboratory-specific long-term reproducibility based on the results of the QC material.
Quality control / Validation	Quality control materials included Duff Brown Tank lacustrine limestone (Hill et al., 2016: 64.04 ± 0.67 Ma), speleothem ASH-15-D-K (Nuriel et al., 2021: 2.965 ± 0.011 Ma) and speleothem ASH-15-C (Vaks et al., 2013: 1.271 ± 0.018 Ma). Data of the QC materials were processed similar to those of the unknowns, i.e. selection and pooling of pixels. The results for the quality control materials are all within uncertainty of the reported reference ages and reported in supplementary data table 3.
**Other information**	All samples were cleaned with ethanol followed by sonication in DIW. Potentially remaining surface contamination was removed during a preablation of all ablated sites. Detailed information on the general analytical protocol and data processing is given in Drost et al. (2018).<br><br>Perach Nuriel, Victor Polyak and Caroline Hill, Nick Roberts kindly provided calcite reference materials ASH, Duff Brown Tank and WC-1, respectively.

References

Chew, D. M., Petrus, J. A., and Kamber, B. S., 2014, U–Pb LA–ICPMS dating using accessory mineral standards with variable common Pb: Chemical Geology, v. 363, p. 185-199, https://doi.org/10.1016/j.chemgeo.2013.11.006

Drost, K., Chew, D., Petrus, J. A., Scholze, F., Woodhead, J. D., Schneider, J. W., and Harper, D. A. T., 2018, An Image Mapping Approach to U-Pb LA-ICP-MS Carbonate Dating and Applications to Direct Dating of Carbonate Sedimentation: Geochemistry, Geophysics, Geosystems, v. 19, no. 12, p. 4631-4648, https://doi.org/10.1029/2018GC007850

Hill, C. A., Polyak, V. J., Asmerom, Y., and P. Provencio, P., 2016, Constraints on a Late Cretaceous uplift, denudation, and incision of the Grand Canyon region, southwestern Colorado Plateau, USA, from U-Pb dating of lacustrine limestone: Tectonics, v. 35, no. 4, p. 896-906, https://doi.org/10.1002/2016TC004166

Horstwood, M. S. A., Košler, J., Gehrels, G., Jackson, S. E., McLean, N. M., Paton, C., Pearson, N. J., Sircombe, K., Sylvester, P., Vermeesch, P., Bowring, J. F., Condon, D. J., and Schoene, B., 2016, Community-Derived Standards for LA-ICP-MS U-(Th-)Pb Geochronology – Uncertainty Propagation, Age Interpretation and Data Reporting: Geostandards and Geoanalytical Research, v. 40, no. 3, p. 311-332, https://doi.org/10.1111/j.1751-908X.2016.00379.x

Jochum, K. P., Weis, U., Stoll, B., Kuzmin, D., Yang, Q., Raczek, I., Jacob, D. E., Stracke, A., Birbaum, K., Frick, D. A., Günther, D., and Enzweiler, J., 2011, Determination of Reference Values for NIST SRM 610–617 Glasses Following ISO Guidelines: Geostandards and Geoanalytical Research, v. 35, no. 4, p. 397-429, https://doi.org/10.1111/j.1751-908X.2011.00120.x

Ludwig, K. R., 2012, User's manual for Isoplot 3.75: Berkley Geochronology Center Special Publication, v. 5, p. 1-75,

Nuriel, P., Wotzlaw, J. F., Ovtcharova, M., Vaks, A., Stremtan, C., Šala, M., Roberts, N. M. W., and Kylander-Clark, A. R. C., 2021, The use of ASH-15 flowstone as a matrix-matched reference material for laser-ablation U − Pb geochronology of calcite: Geochronology, v. 3, no. 1, p. 35-47, https://doi.org/10.5194/gchron-3-35-2021

Paton, C., Hellstrom, J., Paul, B., Woodhead, J., and Hergt, J., 2011, Iolite: Freeware for the visualisation and processing of mass spectrometric data: Journal of Analytical Atomic Spectrometry, v. 26, no. 12, p. 2508-2518, https://doi.org/10.1039/C1JA10172B

Paton, C., Woodhead, J. D., Hellstrom, J. C., Hergt, J. M., Greig, A., and Maas, R., 2010, Improved laser ablation U-Pb zircon geochronology through robust downhole fractionation correction: Geochemistry, Geophysics, Geosystems, v. 11, no. 3, p. Q0AA06, https://doi.org/10.1029/2009gc002618

Petrus, J. A., Chew, D. M., Leybourne, M. I., and Kamber, B. S., 2017, A new approach to laser-ablation inductively-coupled-plasma mass-spectrometry (LA-ICP-MS) using the flexible map interrogation tool 'Monocle': Chemical Geology, v. 463, p. 76-93, http://dx.doi.org/10.1016/j.chemgeo.2017.04.027

Petrus, J. A., and Kamber, B. S., 2012, VizualAge: A Novel Approach to Laser Ablation ICP-MS U-Pb Geochronology Data Reduction: Geostandards and Geoanalytical Research, v. 36, no. 3, p. 247-270, https://doi.org/10.1111/j.1751-908X.2012.00158.x

Roberts, N. M. W., Drost, K., Horstwood, M. S. A., Condon, D. J., Chew, D., Drake, H., Milodowski, A. E., McLean, N. M., Smye, A. J., Walker, R. J., Haslam, R., Hodson, K., Imber, J., Beaudoin, N., and Lee, J. K., 2020, Laser ablation inductively coupled plasma mass spectrometry (LA-ICP-MS) U–Pb carbonate geochronology: strategies, progress, and limitations: Geochronology, v. 2, no. 1, p. 33-61, https://doi.org/10.5194/gchron-2-33-2020

Roberts, N. M. W., Rasbury, E. T., Parrish, R. R., Smith, C. J., Horstwood, M. S. A., and Condon, D. J., 2017, A calcite reference material for LA-ICP-MS U-Pb geochronology: Geochemistry, Geophysics, Geosystems, v. 18, no. 7, p. 2807-2814, https://doi.org/10.1002/2016GC006784

Vaks, A., Woodhead, J., Bar-Matthews, M., Ayalon, A., Cliff, R. A., Zilberman, T., Matthews, A., and Frumkin, A., 2013, Pliocene–Pleistocene climate of the northern margin of Saharan–Arabian Desert recorded in speleothems from the Negev Desert, Israel: Earth and Planetary Science Letters, v. 368, p. 88-100, https://doi.org/10.1016/j.epsl.2013.02.027

Woodhead, J. D., and Hergt, J. M., 2001, Strontium, Neodymium and Lead Isotope Analyses of NIST Glass Certified Reference Materials: SRM 610, 612, 614: Geostandards Newsletter, v. 25, no. 2-3, p. 261-266, https://doi.org/10.1111/j.1751-908X.2001.tb00601.x
